# PKC-ѳ is dispensable for OX40L-induced TCR-independent Treg proliferation but contributes by enabling IL-2 production from effector T-cells

**DOI:** 10.1038/s41598-017-05254-8

**Published:** 2017-07-26

**Authors:** Khaled Alharshawi, Alejandra Marinelarena, Prabhakaran Kumar, Osama El-Sayed, Palash Bhattacharya, Zuoming Sun, Alan L. Epstein, Ajay V. Maker, Bellur S. Prabhakar

**Affiliations:** 1 Department of Microbiology and Immunology, University of Illinois College of Medicine, Chicago, Illinois USA; 2 Department of Immunology, Beckman Research Institute, City of Hope, Duarte, CA USA; 3 Department of Pathology, University of Southern California Keck School of Medicine, Los Angeles, California USA; 4 Department of Surgery, Division of Surgical Oncology, University of Illinois College of Medicine, Chicago, Illinois, USA

## Abstract

We have previously shown that OX40L/OX40 interaction is critical for TCR-independent selective proliferation of Foxp3^+^ Tregs, but not Foxp3^−^ effector T-cells (Teff), when CD4^+^ T-cells are co-cultured with GM-CSF derived bone marrow dendritic cells (G-BMDCs). Events downstream of OX40L/OX40 interaction in Tregs responsible for this novel mechanism are not understood. Earlier, OX40L/OX40 interaction has been shown to stimulate CD4^+^ T-cells through the formation of a signalosome involving TRAF2/PKC-Ѳ leading to NF-kB activation. In this study, using CD4^+^ T-cells from WT and OX40^−/−^ mice we first established that OX40 mediated activation of NF-kB was critical for this Treg proliferation. Although CD4^+^ T-cells from PKC-Ѳ^−/−^ mice were also defective in G-BMDC induced Treg proliferation *ex vivo*, this defect could be readily corrected by adding exogenous IL-2 to the co-cultures. Furthermore, by treating WT, OX40^−/−^, and PKC-Ѳ^−/−^ mice with soluble OX40L we established that OX40L/OX40 interaction was required and sufficient to induce Treg proliferation *in vivo* independent of PKC-Ѳ status. Although PKC-Ѳ is dispensable for TCR-independent Treg proliferation per se, it is essential for optimum IL-2 production by Teff cells. Finally, our findings suggest that OX40L binding to OX40 likely results in recruitment of TRAF1 for downstream signalling.

## Introduction

CD4^+^ T-cell proliferation occurs predominantly through T-cell receptor (TCR) dependent activation and associated co-stimulation^1^. During activation, the TCR binds to its cognate antigen presented through the MHC-II molecule on antigen presenting cells, primarily dendritic cells (DCs), while a second signal is delivered through the co-stimulation of another T cell surface molecule (i.e. CD28) by ligands (like CD80/86) also expressed on the surface of DCs^2^. Both Foxp3^+^ regulatory T cells (Tregs) and Foxp3^−^ effector T cells (Teff) are known to be activated through this common mechanism^3^. Although some studies have shown that low IL-2 concentration and low-strength TCR stimulation can differentially activate Tregs relative to Teff^4, 5^, selective activation of Tregs without affecting Teff is difficult to achieve *in vivo*. While Treg based therapies have been shown to be effective in suppressing autoimmune responses in both mice and humans^6–9^, current clinical approaches require Treg isolation, TCR-based proliferation of Tregs *ex vivo* followed by adoptive transfer^10, 11^. Such an approach limits its potential clinical utility; additionally TCR-based activation methods can also lead to rapid loss of Foxp3 expression and Treg functionality^12^.

We have earlier shown that bone marrow derived dendritic cells (BMDCs) generated *ex vivo* using GM-CSF (G-BMDCs) can selectively cause proliferation of Foxp3^+^ Tregs when co-cultured with total CD4^+^ T-cells^13^. This proliferation was found to be TCR-independent, but critically dependent on OX40 ligand (OX40L) expression on G-BMDCs; and required IL-2 production in those co-cultures by Teff^13^. OX40L is a member of the tumour necrosis factor (TNF) superfamily (TNFSF4), and is the only known ligand for OX40^14^. OX40, a TNF-receptor superfamily (TNFRSF) member (TNFRSF4) also known as CD134^15^ has remained as the only identified receptor for OX40L^16^. It is believed that OX40L/OX40 interaction acts as a co-stimulatory signal during TCR-mediated T-cell activation^17, 18^ and supports prolonged clonal expansion and cytokine secretion^19, 20^. Apart from effector T-cell activation, OX40L/OX40 interaction has been shown to also influence adaptive Treg generation and proliferation^21^, and thymic Treg differentiation^22^. However, these findings have been attributed to OX40L/OX40 mediated co-stimulation in the presence of primary activation signal delivered upon TCR ligation to MHC presented antigens.

The literature on the role of OX40 signalling in TCR-independent activation of T-cells is sparse. Upon ligand binding, TNFR family members including OX40 typically signal through the TNF receptor associated factor (TRAF) family of phylogenetically conserved scaffold proteins^23^ to cause NF-kB activation^24–26^. Different studies have shown association of TRAF1, TRAF2, TRAF3 or TRAF5 with OX40^24, 25, 27–30^. While TCR-associated OX40 signalling can lead to the activation of MAPK, PI3K^31^ and AKT^31, 32^, in the absence of TCR-engagement, ligation of OX40 by OX40L can lead to NF-kB activation through the formation of a “signalosome” containing TRAF2 and protein kinase C-theta (PKC-Ѳ)^33^. PKC-theta (PKC-Ѳ) is a member of the Protein Kinases C (PKC) family which consist of serine/threonine kinases involved in controlling the differentiation and growth of several types of cells^34^. PKC-Ѳ was first cloned from a library of cDNA generated from human peripheral blood lymphocytes^34^. It is expressed in relatively restricted pattern and found primarily in hematopoietic cells^34, 35^. It was found expressed in T-cells, natural killer cells, mast cells, and platelets, but, it was not detected in B-cells, monocytes, macrophages, neutrophils, and erythrocytes^35–38^. The expression of PKC-Ѳ in T-cells vary based on homing organ, it is high in the thymus and lymph nodes, low in the spleen, and undetectable in bone marrow^35–37^. PKC-Ѳ is known to play a critical role as part of the “immunological synapse,” at the interface of antigen presenting cells and T-cells that is required for TCR-mediated T-cell activation^39, 40^. Because of its role in T-cell activation and function^41–43^, PKC-Ѳ is targeted for developing novel immunosuppressive regimens^44, 45^. In contrast to the above mentioned immune-stimulatory role, PKC-Ѳ^−/−^ mice exhibit low Treg frequency in the periphery^46^; possibly a consequence of an intrinsic defect in thymic Treg development and/or a Treg extrinsic defect in IL-2 production by Teff^46, 47^. However, if PKC-Ѳ was indeed involved in TCR-independent Treg proliferation, it would point to an alternative mechanism behind the low Treg numbers found in PKC-Ѳ deficient mice.

In the present work, we investigated whether the underlying mechanism of TCR-independent proliferation of Tregs observed in *ex vivo* G-BMDCs/T-cell co-cultures involved OX40-dependent activation of NF-kB through formation of TRAF2/PKC-Ѳ signalosome. First, by co-culturing T-cells from OX40^−/−^ mice with G-BMDCs, we confirmed the critical role of OX40L/OX40 interaction in TCR-independent Treg proliferation. Further, using soluble OX40L treatment, we established that OX40L/OX40 interaction was sufficient to cause functional Treg proliferation *in vivo*. Next, we demonstrated that TCR-independent Treg proliferation likely involved OX40 association with TRAF1, leading to NF-kB activation and cell cycle progression. Further, using T-cells from PKC-Ѳ^−/−^ mice we established that PKC-Ѳ was primarily required for IL-2 production by Teff, which was needed for Treg survival. Proliferation of PKC-Ѳ^−/−^ Tregs upon supplementation with exogenous IL-2 suggested that expression of PKC-Ѳ per se was dispensable for TCR-independent Treg proliferation.

## Results

### OX40L/OX40 interaction is indispensable for G-BMDC-induced *ex vivo* expansion of Tregs

In earlier studies we have shown that GM-CSF treatment can prevent the development of autoimmune diseases in multiple mouse models^48–52^. The therapeutic effect of GM-CSF treatment was mediated through the mobilization of tolerogenic dendritic cells^48^, which caused the proliferation of Tregs that suppressed the autoimmune disease via increased IL-10 production^53^. Further, we have shown that GM-CSF derived bone marrow DCs (G-BMDCs) can induce robust proliferation of Foxp3^+^ Tregs in *ex vivo* co-cultures with CD4^+^ T-cells^13^. Additionally, we had found that OX40L/OX40 interaction was critical for this Treg proliferation^13^.

**Figure 1:**
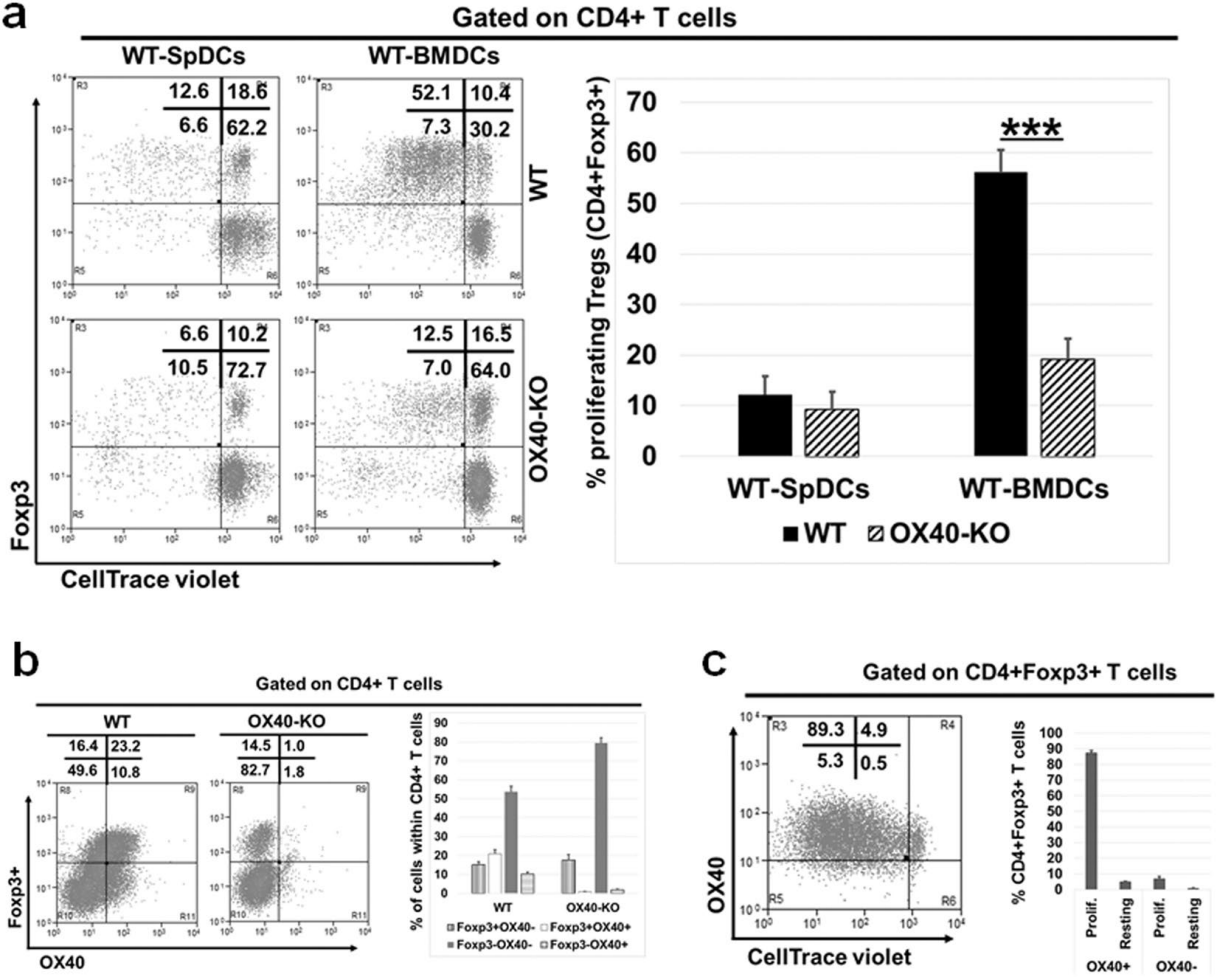
OX40-KO Tregs show impaired proliferation compared to WT Tregs. WT and OX40^−/−^splenic total CD4^+^ T-cells, isolated and labelled with CellTrace violet, were co-cultured with WT-BMDCs. After five days, cells were harvested and analysed by flow cytometry. (**a**) Dot plot (left) and summarizing bar graphs (right) show Treg proliferation in OX40-KO cells compared to WT. (**b**) Dot plot (left) and summarizing bar graphs (right) showing the expression of Foxp3 and OX40 in WT and OX40-KO CD4+ T-cells.  (**c**) Dot plot (left) and summarizing bar graphs (right) showing the expression of OX40 among the proliferating and resting Tregs (CD4^+^Foxp3^+^). Values show average ± SD, *P < 0.05, **p < 0.005, and ***p < 0.0005.

In this study, we wanted to further explore the specific role of OX40L/OX40 interaction in G-BMDC mediated selective Treg proliferation. First we co-cultured splenic CD4^+^ T-cells isolated from WT and OX40^−/−^ mice with G-BMDCs generated from WT bone marrow progenitors and measured the proliferation of Tregs (Fig. 1a). We observed a significant reduction (p < 0.0005) in the proliferation of OX40^−/−^ Tregs compared to WT Treg counterparts (Fig. 1a). Next we analysed the expression of OX40 which showed, as expected, the absence of OX40 expression on CD4^+^ T-cells derived from OX40^−/−^ (Fig. 1b). Further, we observed that almost all the proliferating WT Tregs were OX40^+^ (Fig. 1c). These results confirmed our earlier studies that OX40 signalling is a critical requirement in G-BMDC-induced Treg proliferation.

Previous studies have reported a negative effect of OX40L on Foxp3 expression^54, 55^. However, in our study we did not find any reduction in Foxp3 expression in Tregs expanded with OX40L either *in vitro* or *in vivo* (Supplementary Fig. S1). Next we analysed whether proliferating Tregs retained their suppressive phenotype. We characterized the expression of CTLA4, Eos, CD39 and CD44 which typically determine the suppressive phenotype of Tregs^56–62^. When compared to the resting Tregs, a higher percentage of proliferating Tregs expressed these markers (Supplementary Fig. S2). These results showed that Tregs expanded by G-BMDCs maintained their suppressive phenotype and thus likely their suppressive function.

### OX40L treatment increases functional Treg numbers *in vivo* and reduces insulitis in NOD mice

**Figure 2:**
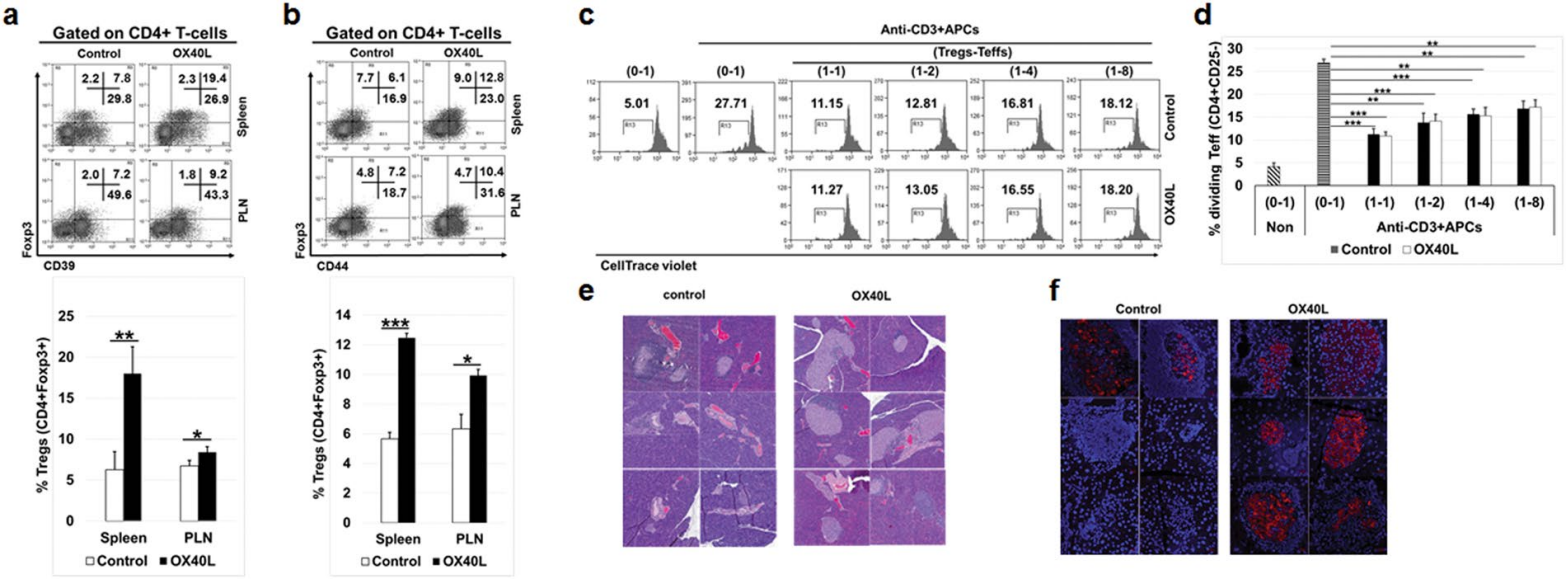
OX40L treatment leads to expansion of functional Tregs *in vivo*. NOD mice were injected i.p. with either PBS or OX40L three times (200 µg/mouse/week). One week after the last treatment, mice were euthanized and cells were collected for analysis. (**a**) Representative dot plots (left) and bar graphs (right) show Foxp3 and CD39 expression in spleen and PLN. (**b**) Representative dot plots (left) and bar graphs (right) show Foxp3 and CD44 expression in spleen and PLN. (**c**,**d**) Splenic Teffs and Tregs were isolated from the untreated and treated mice and used in an *ex vivo* suppression assay. (**c**) Representative histograms show results from the *ex vivo* suppression assay. (**d**) Bar graphs summarizing results shown in C. Values show average ± SD, *P < 0.05, **p < 0.005, and ***p < 0.0005. (**e**) H&E stained pancreatic tissue sections from untreated and OX40L-treated NOD mice showing different degrees of lymphocytic infiltration and islet damage at 23 weeks of age. (**f**) Immunofluorescence microscopy of pancreatic tissue sections stained with anti-insulin antibody.

To demonstrate that this mechanism of Treg proliferation is conserved *in vivo*, we treated 6-week old NOD mice with three doses (dose/week) of either OX40L or PBS. After the fourth week, we analysed for the expression of suppressor function markers on Tregs. OX40L treated NOD mice showed a substantial increase in Tregs that were positive for CD39 (Fig. 2a), a marker of Treg function^59, 60^, and CD44 (Fig. 2b), a marker of Treg stability and function^61, 62^. In order to evaluate the suppressive function of *in vivo* expanded Tregs, CD4^+^CD25^−^ T-cells isolated from untreated diabetic NOD mice were labelled with CellTrace violet and cultured in the presence of splenic APCs with and without stimulation with anti-CD3. To these cultures, we added Tregs from the untreated control or OX40L treated mice in different ratios (Fig. 2c and d). Tregs from OX40L treated mice showed suppressive function that was comparable to Tregs isolated from untreated control mice (Fig. 2c and d). Collectively these results suggested that OX40 signalling *in vivo* can expand Tregs without the loss of their suppressive function. Furthermore, when we analysed pancreatic sections of treated and untreated mice at 23 weeks of age by H&E staining, we found many intact islets in the OX40L treated group while most of the islets from untreated mice were heavily infiltrated with lymphocytes (Fig. 2e). Additionally, we observed many insulin positive islets in the pancreatic sections from OX40L treated mice while such islets were sparse in the untreated group (Fig. 2f). Thus, the increase in Tregs *in vivo* upon OX40L treatment was also functionally correlated with reduced lymphocyte migration to pancreatic islets and consequently lower islet loss.

### Critical involvement of NF-kB in G-BMDC-induced *ex vivo* Treg proliferation

**Figure 3:**
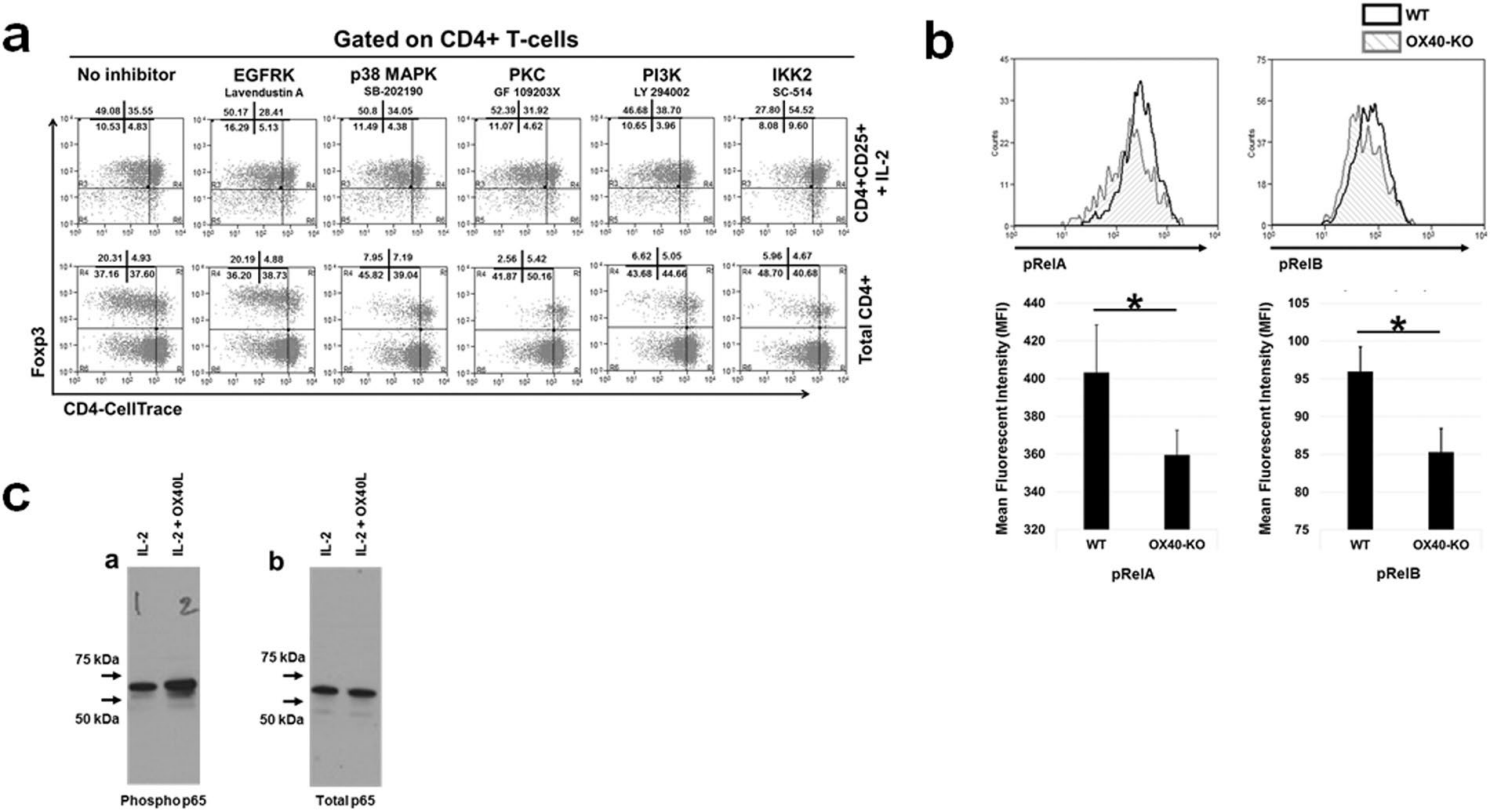
Signalling pathways involved in Treg proliferation induced by G-BMDCs. (**a**) Total CD4^+^ and CD4^+^CD25^+^ T-cells isolated from NOD mice spleen were CellTrace violet-labelled, incubated for 6 hrs in the presence of various kinase inhibitors, and then co-cultured with WT-BMDCs in the absence (total CD4+ T cells) or presence (CD4^+^CD25^+^ T-cells) of exogenous IL-2 (5 U/ml). Cells were harvested on day 5 and analysed by flow cytometry for Treg proliferation. Representative dot plots for Treg proliferation are shown for indicated inhibitors. (**b**) WT and OX40-KO splenic CD4+ T-cells were isolated, labelled with CellTrace violet and co-cultured with MHC-class II-KO-BMDCs in presence of exogenous IL-2 (1 U/ml). After 5 days, cells were harvested and analysed by flow cytometry for activation of NF-kB pathway. Mean fluorescence intensity (MFI) of the indicated molecules are shown in bar graphs. (**c**) CD4^+^ T-cells isolated from splenocytes were treated with IL-2 alone or with IL-2 and OX40L for 24 h. Cells were lysed and analysed for NF-kB-p65 phosphorylation using total and phospho-p65 antibodies by western blot. Values show average ± SD, *P < 0.05, **p < 0.005, and ***p < 0.0005.

Depending on the presence or absence of TCR signalling, it is known that OX40 can act as a co-stimulatory molecule or as an independent receptor, and activate different down-stream signals mediated by different kinases including MAPK and PI3K^31, 63^. To understand which particular signalling pathways were involved in the OX40 dependent proliferation of Tregs, we incubated either total CD4^+^ T cells or CD4^+^CD25^+^ Tregs with kinase inhibitors targeting the EGFRK, p38 MAPK, PKC, PI3K and IKK2 (NF-kB) signalling pathways. Subsequently, we co-cultured G-BMDCs with these kinase inhibitor exposed and CellTrace violet labelled CD4^+^ T cells and Tregs supplemented with IL-2, and measured the extent of cell proliferation using flow cytometry (Fig. 3a). We found reduction in Treg proliferation upon inhibition of IKK2 kinase, in both CD4^+^ T-cell as well as CD4^+^CD25^+^ Treg co-cultures supplemented with IL-2. These results suggested that activation of the NF-kB signalling pathway is critical for G-BMDC mediated Treg proliferation.

To elucidate the importance of NF-kB signalling in the TCR-independent proliferation of Tregs, we analysed the phosphorylation of RelA and RelB, signalling molecules involved in NF-kB heterodimer formation in Tregs after co-culture with G-BMDCs. We co-cultured WT and OX40^−/−^ CD4^+^ T-cells with MHC class II^−/−^ G-BMDCs in the presence of exogenous IL-2. After five days (Fig. 3b), cells were harvested and analysed by flow cytometry for the extent of phosphorylation of RelA and RelB by comparing their mean fluorescence intensities (MFI). The MFIs of pRelA, and pRelB were significantly (p < 0.05 for both) up-regulated in the WT Tregs suggesting NF-kB activation upon OX40L signalling (Fig. 3b). We also confirmed increased phosphorylation of NF-kB p65 (RelA) upon soluble OX40L/IL-2 co-treatment of WT CD4^+^ T-cells *ex vivo* by Western blot (Fig. 3c). These data collectively suggested that OX40 activation even in the absence of TCR-stimulation leads to the activation of NF-kB in Tregs, which in turn may be critical for Treg proliferation.

### PKC-Ѳ^−/−^ T-cells exhibit defective Treg-proliferation in G-BMDC co-cultures

**Figure 4:**
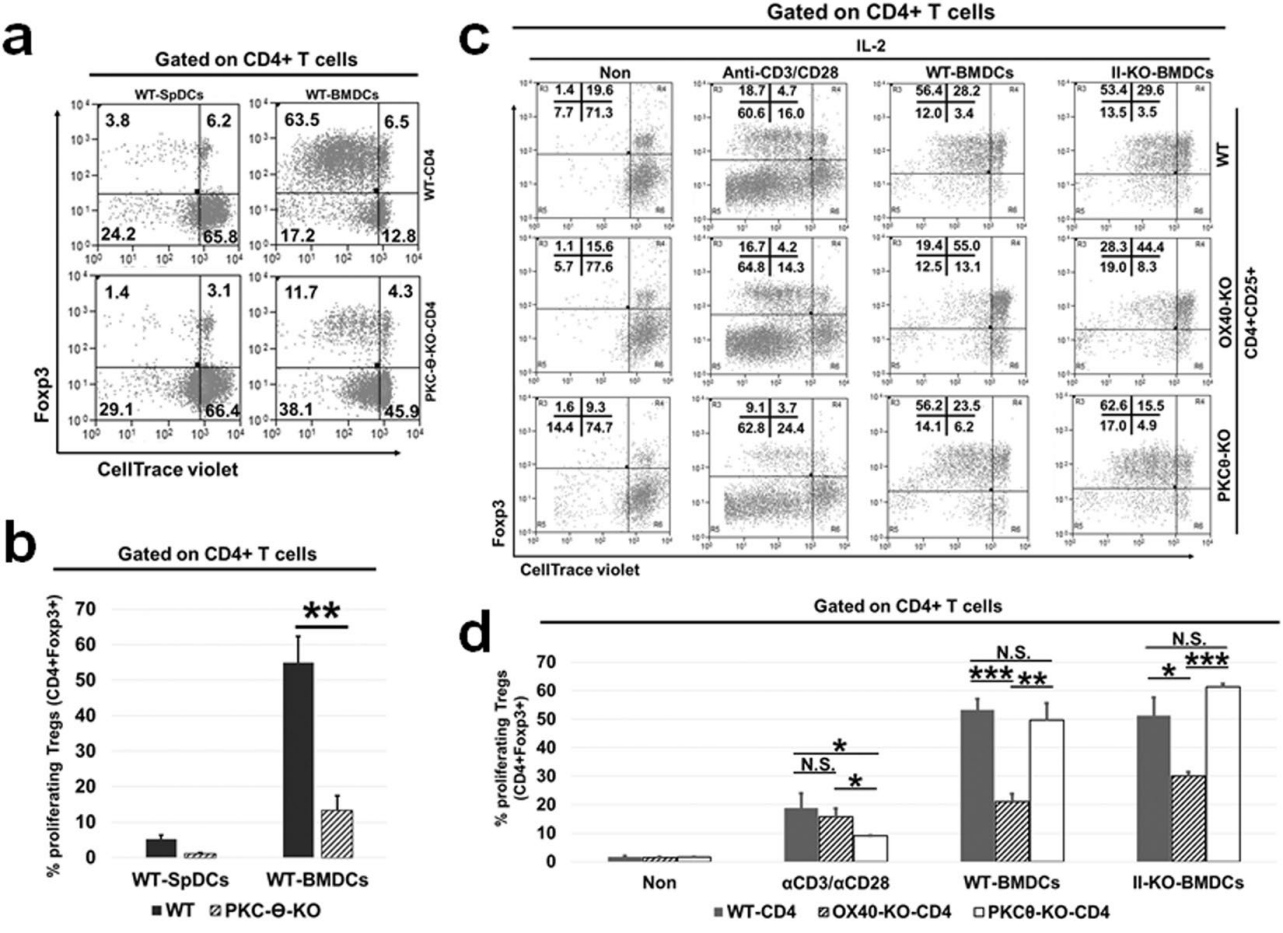
PKC-Ѳ-KO total CD4^+^ T-cells when co-cultured with WT-BMDCs showed a defect in Treg proliferation. (**a**,**b**) Total CD4^+^ T-cells were isolated from the spleens of WT and PKC-Ѳ-KO mice, CellTrace violet-labelled, then co-cultured with WT-BMDCs. Cells were harvested on day 5 and analysed by flow cytometry for Treg proliferation. (**a**) Representative dot plots (**b**) summary bar graphs of percentage of proliferating Tregs. (**c**,**d**) Splenic CD4^+^CD25^+^ T-cells isolated from WT, PKCѲ-KO and OX40-KO mice, then cultured in the presence of exogenous IL-2, with microbeads coated with anti-CD3/anti-CD28 or WT/Class-II-KO-BMDCs. Cells were harvested after 4 days and analysed by flow cytometry. Dot plots (**c**) showing the proliferation of Tregs and bar graphs (**d**) representative of data shown in (**c**). Numbers shown in the dot plot is the average percentage (n = 3). Values show average ± SD, *P < 0.05, **p < 0.005, and ***p < 0.0005.

It has been suggested that OX40 activation upon OX40L binding, in the absence of TCR-engagement, is capable of up-regulating NF-kB through the formation of a “signalosome” consisting of TRAF2 and PKC-Ѳ^33^. Therefore, PKC-Ѳ has been thought to be a critical component of the “signalosome” involved in OX40 mediated signalling independent of TCR-activation. On account of these earlier findings, we investigated the role of PKC-Ѳ in G-BMDC mediated Treg proliferation. Total CD4^+^ T cells were isolated from the spleens of PKC-Ѳ^−/−^ mice and co-cultured with WT G-BMDCs to measure Treg proliferation (Fig. 4a and b). While WT Tregs proliferated robustly, PKC-Ѳ^−/−^ Tregs exhibited weak proliferation (55 ± 7.4% vs 13.2 ± 4.2%), suggesting a possible role for PKC-Ѳ in G-BMDC mediated Treg proliferation (Fig. 4a and b).

### Exogenous IL-2 restores proliferation of PKC-Ѳ^−/−^ Tregs in G-BMDC co-cultures

We have previously shown that MHC class II^−/−^ G-BMDCs were capable of inducing Treg proliferation; however, this proliferation was dependent on the presence of exogenous IL-2^13^. To see if a deficit in IL-2 production was responsible for the substantially reduced proliferation of PKC-Ѳ^−/−^ Tregs, we isolated CD4^+^CD25^+^ Tregs from WT, PKC-Ѳ^−/−^, and OX40^−/−^ mice, and co-cultured these cells separately with either anti-CD3/anti-CD28 coated beads, WT G-BMDCs, or MHC class II^−/−^ G-BMDCs in the presence of exogenous IL-2 and measured Treg proliferation (Fig. 4c and d). We observed comparable levels of proliferation of both OX40^−/−^ and WT Tregs when stimulated with anti-CD3/antiCD28 suggesting that these cells were competent for TCR-dependent proliferation (Fig. 4c and d). However, when co-cultured with either WT G-BMDCs or MHC class II^−/−^ G-BMDCs, OX40^−/−^ Tregs showed significantly (p < 0.0005, p < 0.05, respectively) reduced proliferation compared to WT Tregs (Fig. 4c and d). These results suggested that OX40 signalling is indispensable for the TCR independent, and not for the TCR-dependent, Treg proliferation. Further, this requirement could not be rectified by the addition of exogenous IL-2. In contrast, proliferation was restored to WT levels in PKC-Ѳ^−/−^ Tregs when co-cultured with either WT G-BMDCs or MHC class II^−/−^ G-BMDCs supplemented with IL-2 (Fig. 4c and d).

**Figure 5:**
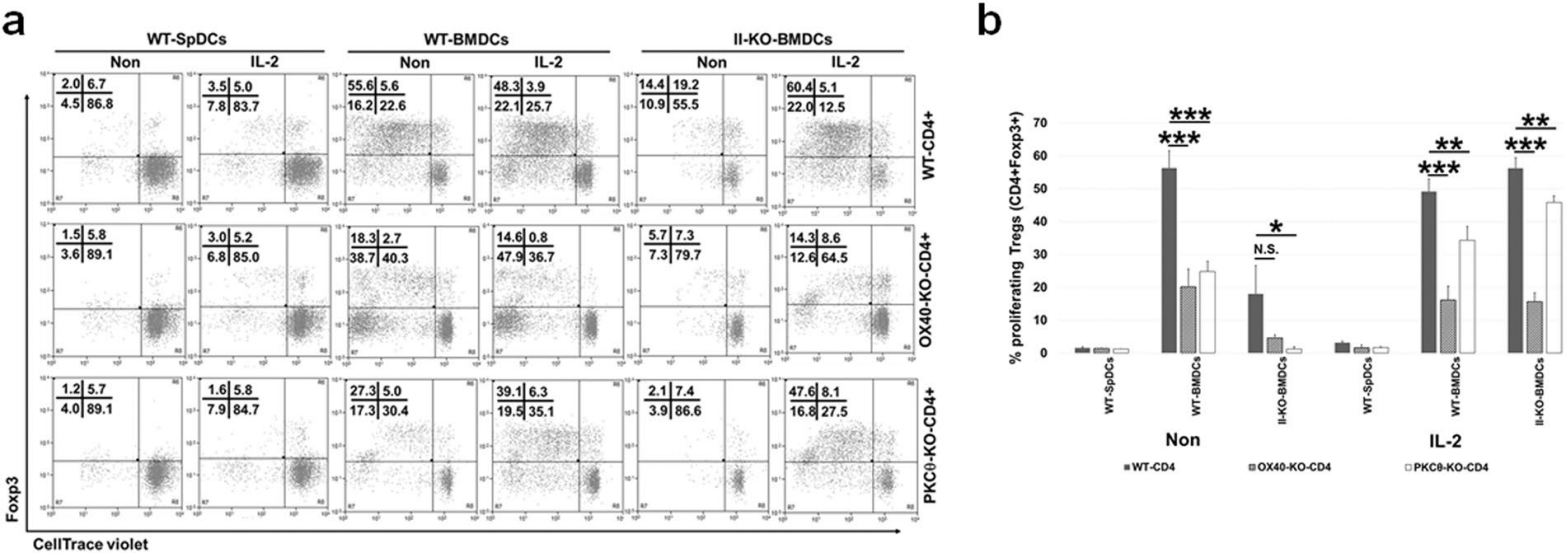
Exogenous IL-2 rescues the Treg proliferation phenotype when added to co-cultures containing WT and PKCθ-KO not OX40-KO CD4^+^ T-cells. Total WT, OX40-KO and PKCθ-KO splenic CD4^+^ T-cells, isolated and CellTrace-labelled, were co-cultured with either WT-BMDCs or II-KO-BMDCs in the presence or absence of exogenous IL-2. After 5 days, cells were harvested and analysed by FACS. (**a**) Representative dot plots show the proliferating Tregs upon co-culture of CD4^+^ T-cells. (**b**) Bar graphs comparing the percentage of proliferating Tregs from OX40-KO and PKCθ-KO to WT CD4^+^ T-cells, in various co-culturing conditions. Values show average ± SD, *P < 0.05, **p < 0.005, and ***p < 0.0005.

Next, we analysed whether PKC-Ѳ^−/−^ T-cells have defective IL-2 production. As shown in supplementary Fig. S3, PKC-Ѳ^−/−^ T-cells showed impaired IL-2 production compared to WT-T-cells. In order to further investigate the role of IL-2 in PKC-Ѳ^−/−^ Treg proliferation, we co-cultured total CD4^+^ T-cells from WT, OX40^−/−^, and PKC-Ѳ^−/−^ mice with WT G-BMDCs or MHC class II^−/−^ G-BMDCs in the presence or absence of exogenous IL-2. As expected, the WT CD4^+^ T-cells proliferated efficiently and comparably when co-cultured with WT-G-BMDCs in the presence or absence of exogenous IL-2 (Fig. 5a and b). Additionally, when co-cultured with MHC class II^−/−^ G-BMDCs, WT CD4^+^ T-cells showed poor proliferation, which was restored upon the addition of IL-2 (Fig. 5a and b). OX40^−/−^ CD4^+^ T-cells co-cultured with either WT G-BMDCs or MHC class II^−/−^ G-BMDCs showed impaired Treg proliferation compared to WT Tregs both in the presence or absence of IL-2 (Fig. 5a and b). This supported our previous results and suggested that OX40 signalling is critically required for G-BMDC-induced Treg proliferation. On the contrary, we observed partially (24.8 ± 3.1% vs 56.3 ± 5.2% in WT) and fully (1.2 ± 0.7% vs 18.0 ± 8.6% in WT) impaired PKCѲ^−/−^ Treg proliferation in co-cultures with WT G-BMDCs or MHC class II^−/−^ G-BMDCs respectively. Interestingly, PKC-Ѳ^−/−^ Treg proliferation was largely restored in both co-cultures upon supplementation with exogenous IL-2 (Fig. 5a and b). These data suggested that PKC-Ѳ is not critically required as a downstream partner of OX40 mediated signalling for Treg proliferation, but may be required for IL-2 production. Collectively, these data suggested that while OX40 signalling plays a critical and direct role in TCR-independent Treg proliferation, PKC-Ѳ may play an indirect role by facilitating IL-2 production, likely by Teff cells.

### PKC-Ѳ is required for IL-2 production by Teff cells, which is critical for Treg proliferation in G-BMDC co-cultures

**Figure 6:**
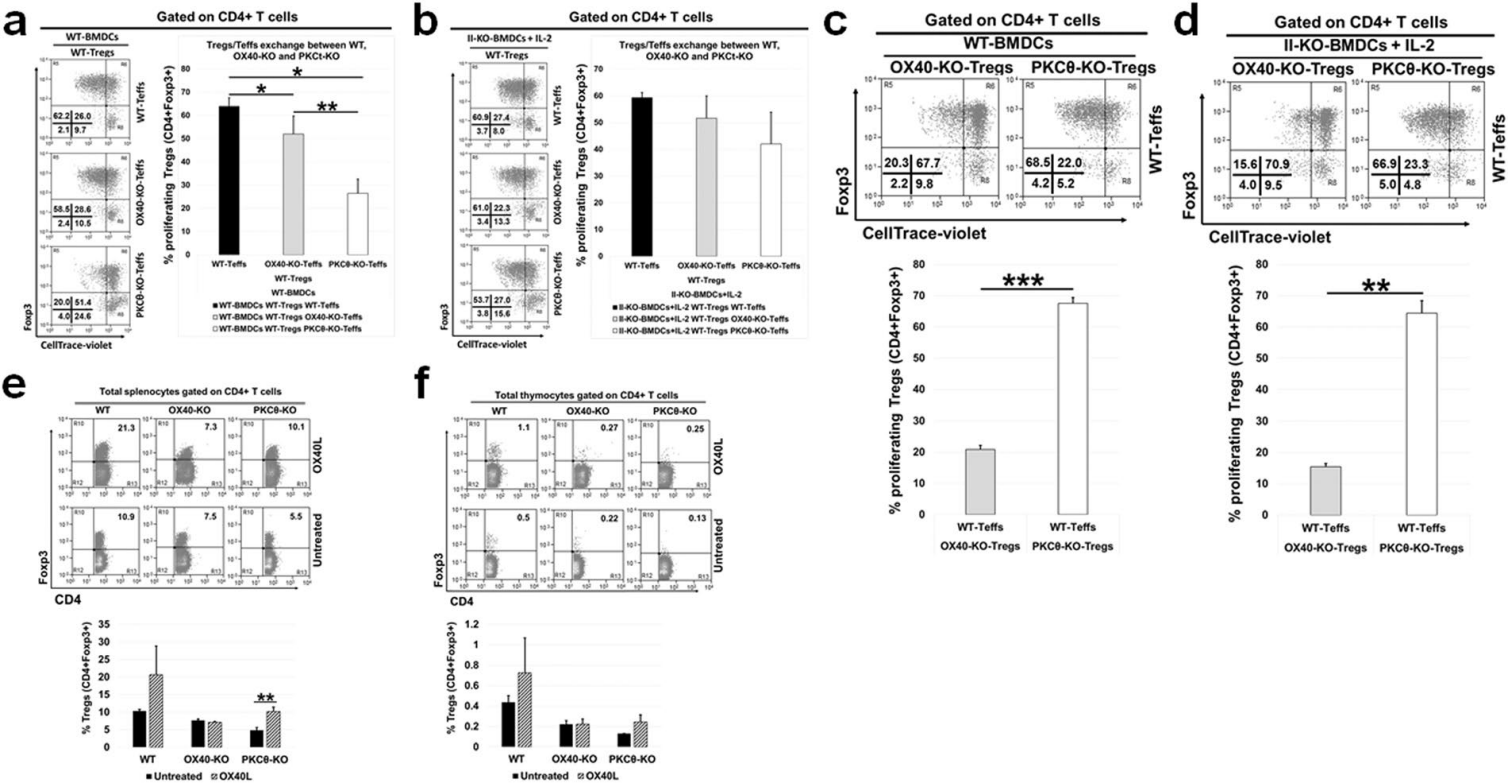
PKCθ-KO Treg proliferation, but not OX40−/−, phenotype can be rescued when cultured with WT Teffs. (**a**–**d**) Splenic Tregs (CD4^+^CD25^+^) isolated from WT, OX40-KO, and PKCθ-KO mice were labelled with CellTrace violet and then each Treg subtype (WT, OX40-KO & PKCθ-KO) was remixed with the Teffs (CD4^+^CD25^−^) from their own counterpart and the other two cell subtypes as indicated. Subsequently, these T-cell combinations were co-cultured with WT-BMDCs or II-KO-BMDCs+ IL-2 (1 U/ml) for 4 days then harvested and analysed by flow cytometry. (**a**) Representative dot plots (left) and summarizing bar graphs (right) show the percentage of proliferating Tregs when WT Tregs were remixed with either WT Teffs, OX40-KO Teffs or PKC-θ-KO Teffs then co-cultured with WT-BMDCs. (**b**) Representative dot plots (left) and summarizing bar graphs (right) show the percentage of proliferating Tregs when WT Tregs were remixed with either WT Teffs, OX40-KO Teffs or PKC-θ-KO Teffs then co-cultured with II-KO-BMDCs. (**c**) Representative dot plots (top) and summarizing bar graphs (bottom) show the percentage of proliferating Tregs when OX40-KO or PKC-θ-KO Tregs remixed with WT Teffs then co-cultured with WT-BMDCs. (**d**) Representative dot plots (top) and summarizing bar graphs (bottom) show the percentage of proliferating Tregs when OX40-KO or PKC-θ-KO Tregs remixed with WT Teffs then co-cultured with II-KO-BMDCs. E&F) WT, OX40-KO, and PKCθ-KO mice were treated with soluble OX40L three times (100 µg/mouse/week). Another group of mice received PBS (untreated control). One week after the last treatment, mice were euthanized and spleen and thymus were collected for analysis by flow cytometry. (**e**) Representative dot plots show the percentage of Tregs from total CD4^+^ splenocyte T-cells (left) and summarizing bar graphs (right). (**f**) Representative dot plots show the percentage of Tregs from total CD4^+^ thymocyte T-cells (left) and summarizing bar graphs (right). Values showing average ± SD, *P < 0.05, **p < 0.005, and ***p < 0.0005.

To investigate the hypothesis that PKC-Ѳ is required for the production of IL-2 by Teffs which in turn is required for the proliferation of Tregs, we co-cultured Tregs and Teffs from WT, OX40^−/−^ and PKC-Ѳ^−/−^ mice in various combinations and measured Treg proliferation. First, we isolated CD4^+^CD25^+^ (Tregs) and CD4^+^CD25^−^ (Teffs) T cells from WT, OX40^−/−^ and PKC-Ѳ^−/−^ mice. Subsequently, WT Tregs, labelled with CellTrace violet, were co-cultured with Teffs from WT, OX40^−/−^ or PKC-Ѳ^−/−^ mice in the presence of WT-BMDCs. In the presence of OX40^−/−^ Teff cells, WT Tregs proliferated to a level comparable to that with WT Teffs (Fig. 6a). However, WT Treg proliferation was significantly (p < 0.05) reduced when co-cultured with PKC-Ѳ^−/−^ Teffs relative to when co-cultured with WT Teffs. These results suggested a role for PKC-Ѳ in IL-2 production by Teffs (Fig. 6a). To further establish that the primary defect in PKC-Ѳ^−/−^ Teffs was in IL-2 production we cultured MHC-II^−/−^ BMDCs and WT Tregs, with Teffs from WT, OX40^−/−^, or PKC-Ѳ^−/−^ mice in the presence of exogenous IL-2. As expected, no defect in Treg proliferation was observed when MHC-II^−/–^BMDCs and WT-Tregs were co-cultured with OX40^−/−^ Teffs. Furthermore, MHC-II^−/−^ BMDCs and WT Tregs co-cultured with PKC-Ѳ^−/−^ Teffs and supplemented with IL-2 also did not exhibit a defect in Treg proliferation, supporting a primary role of PKC-Ѳ in IL-2 production by Teffs (Fig. 6b).

In complementary experiments, to evaluate the competence of PKC-Ѳ^−/−^ Tregs in TCR-independent proliferation, WT Teffs were co-cultured with Tregs isolated from OX40^−/−^ or PKC-Ѳ^−/−^ mice in the presence of either WT BMDCs (or MHC-II^−/−^ BMDCs in the presence of IL-2) and Treg proliferation was measured (Fig. 6c and d). In the presence of WT Teffs, PKC-Ѳ^−/−^ Tregs proliferated to WT Treg levels either in the presence of WT BMDCs (Fig. 6c) or MHC-II^−/−^ BMDCs/IL-2 (Fig. 6d). This established that PKC-Ѳ^−/−^ Tregs are intrinsically competent for TCR-independent proliferation, so long as the Teffs are capable of secreting IL-2. However, OX40^−/−^ Tregs co-cultured in the presence of WT Teffs and WT G-BMDCs showed a substantially lower Treg proliferation compared to that of PKC-Ѳ^−/−^ Tregs. This defect in OX40^−/−^ Treg proliferation was also observed when OX40^−/−^ Tregs were cultured in the presence of WT Teffs and MHC-II^−/−^ BMDCs with IL-2 supplementation (Fig. 6d). These results further suggested that requirement of OX40 signalling is intrinsic to, and indispensable for, the *ex vivo* proliferation of Tregs.

### PKC-Ѳ is dispensable for OX40L-mediated *in vivo* Treg expansion

To investigate the requirement of OX40 signalling *in vivo* and further test the requirement of PKC-Ѳ, we administered OX40L three times (once/week) intraperitoneally (i.p.) to WT, OX40^−/−^ and PKC-Ѳ^−/−^ mice and measured the percentages of Tregs one week after the last treatment (Fig. 6e and f). With OX40L treatment, WT and PKC-Ѳ^−/−^ mice showed an increase in the percentage of Tregs within the CD4^+^ splenocyte population when compared to their untreated counterparts (Fig. 6e). On the other hand, no change in Treg percentage was observed in OX40L-treated OX40^−/−^ mice (Fig. 6e). While we observed an increase in the percentage of Tregs within the CD4^+^ thymocytes from OX40L treated WT and PKC-Ѳ^−/−^ mice compared to their untreated counterparts, we failed to see a similar increase in Tregs in OX40L treated OX40^−/−^ mice (Fig. 6f). These results are consistent with our *ex vivo* data and suggested that OX40L-induced Treg proliferation did not critically require PKC-Ѳ for OX40 mediated signalling.

### OX40 likely associates with TRAF1 in a Treg specific manner to drive TCR-independent Treg proliferation

**Figure 7:**
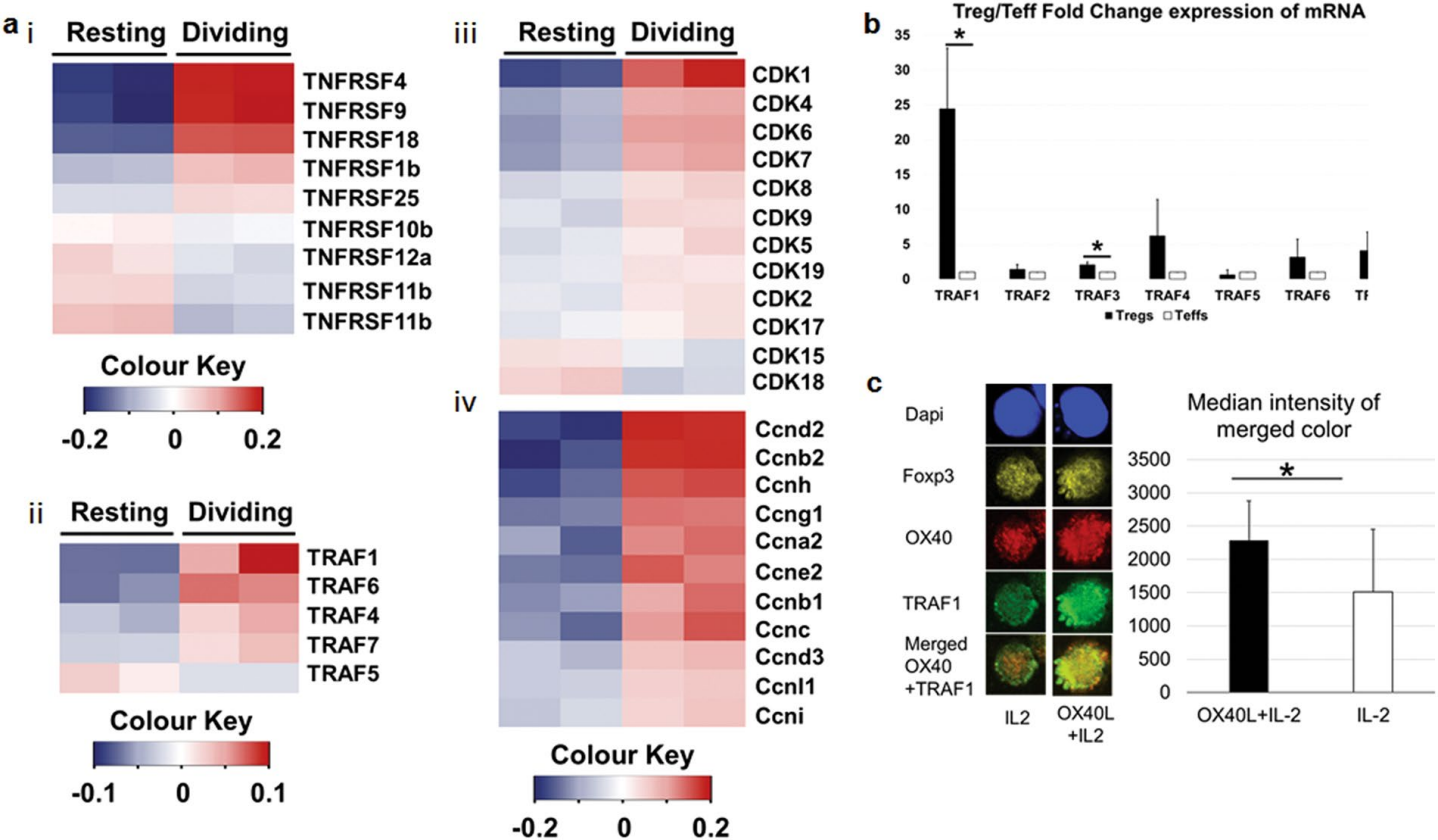
OX40 interaction with TRAF1 is likely involved in Treg proliferation. (**a**) Gene-array analysis of fold difference in mRNA expression between proliferating and resting Tregs. Heat maps showing positive fold-difference in red under phenotypic/functional categories including i) TNF-family receptors, ii) TRAFs, iii) CDKs and iv) cyclins. (**b**) TRAF1 is highly upregulated in Tregs compared to Teffs. Splenic Tregs (CD4+CD25+ T cells) and Teffs (CD4+CD25− T cells) were isolated. cDNA was synthesized from mRNA isolated from Tregs and Teffs and subjected to RT-PCR. Ct is used to calculate the fold change in TRAFs expression in Tregs over Teffs. (**c**) Co-localization of OX40 and TRAF1 upon treatment with OX40L. Splenic total CD4+ T cells isolated from wild type B6 mice, cultured in the presence of IL-2 with or without soluble OX40L. After 48 hrs cells were harvested and analysed by confocal microscopy. Representative images (left) and bar graphs summarizing the median intensity of merged colour (right) between OX40 (red) and TRAF1 (green) in OX40L+ IL-2 vs IL-2 treated. Values showing average ± SD, *P < 0.05, **p < 0.005, and ***p < 0.0005.

In our recent studies we have used microarray analysis to compare gene expression differences between G-BMDC directed proliferating Tregs and resting Tregs^64^ (NCBI-GEO; Accession No. GSE81051). Upon further analysis of gene array data (Fig. 7a), we observed strong upregulation of OX40, 4-1BB, and GITR mRNA (with moderate upregulation of TNFR2) amongst members of the TNFR super family, in proliferating versus resting Tregs (Fig. 7a i). The signalling via TNF-receptors in general and OX40 in particular has been shown to induce the formation of a signalosome which subsequently activates NF-kB pathway. The main components of the signalosome are the TNF-receptor associated factor (TRAF) family members (TRAF1-7). Among the TRAFs, we found maximum elevation in the expression of TRAF1 mRNA in the proliferating Tregs (Fig. 7a ii). Among the cell cycle related molecules we found up-regulation of CDK1 (Fig. 7a iii) and cyclin D2 (Fig. 7a iv) mRNAs in proliferating Tregs. NF-kB has been shown to activate cyclin D2 to promote cell cycle progression in resting T-cells^65^. Further, CDK1 has been shown to be able to bind to all cyclins (including Cyclin D2) and appears to be capable of driving cell cycle progression in the absence of other interphase CDKs^66^. Thus, OX40 may associate with TRAF1 to activate NF-kB signalling, leading to cyclin D2 synthesis and cell proliferation in Tregs.

To further investigate the involvement of TRAF1 in Treg proliferation, we first used real time PCR to determine if TRAF1 was preferentially expressed in Tregs compared to Teff. We isolated total RNA from Tregs (CD4^+^CD25^+^) and Teff (CD4^+^CD25^−^) from the spleens of wild type mice, synthesized cDNA and conducted real-time PCR using primers for mouse TRAF1-7 (Supplementary Table S1). Interestingly, TRAF1 was found to be significantly (p < 0.05) upregulated in Tregs compared Teff (Fig. 7b), which is also consistent with similar observation in human Tregs^67^.

To evaluate if TRAF1 was associated with OX40 in Tregs upon OX40L stimulation, we isolated total splenic CD4^+^ T cells and cultured them in the presence of IL-2 with or without OX40L. Subsequently, we stained the cells for TRAF1, OX40, and Foxp3, and analysed them by confocal microscopy. We found that Tregs (Foxp3^+^) exposed to OX40L showed increased co-localization of TRAF1 and OX40 compared to Tregs which did not receive OX40L (Fig. 7c). These data suggested that TRAF1 is likely involved in OX40-downstream signalling leading to NF-kB activation and proliferation of Tregs.

## Discussion

Dendritic cells play a critical role in regulating innate and adaptive immunity including regulatory and effector T-cell responses. DCs express a wide array of co-stimulatory molecules which facilitate antigen presentation and T-cell expansion. However a subset of DCs namely, tolerogenic DCs have been shown to promote immune-tolerance by expanding Tregs which can suppress effector T-cell responses^48–50^. OX40L is a co-stimulatory molecule expressed on a subset of DCs which can enhance T-cell activation and effector function upon TCR activation. Additionally, OX40L expressing DCs can enhance Th2 cell differentiation from naïve T-cells and promote Th2 responses^68–70^. Consistent with this, in a recent study, OX40L expressing monocyte derived DCs have been reported to bridge human B-cell and T-cell communication and promote Th2 cell expansion^71^. In our earlier study, we found increased Tregs in NOD mice treated with soluble OX40L and Jagged-1 and also observed increased Th2 cytokine expression upon TCR stimulation *in vitro* ^64^. Interestingly, naïve Tconv cells do not express OX40 and require TCR activation to gain transient OX40 expression. Therefore, upon TCR activation OX40L might drive Th2 polarization and proliferation of activated Teff cells. In contrast, under steady state conditions, we found Tregs constitutively expressing OX40 and identified a unique subset of bone marrow derived OX40L^+^DCs which are capable of selectively expanding Tregs in a TCR-independent manner without affecting effector T-cell proliferation^13^.

There are two unique features of our finding of G-BMDC mediated Treg proliferation that sets it apart from canonical TCR-dependent T-cell activation and proliferation^1^. Firstly, it differentially drives the proliferation of Foxp3^+^ Tregs. Secondly, this proliferation is independent of MHC-II/TCR interaction. We had earlier identified OX40L expressed by G-BMDCs to be a critical ligand in this G-BMDC/Treg interaction. OX40L signals through its cognate receptor OX40, which is constitutively expressed on Tregs^72^. Since IL-2 acts as an indispensable survival factor for Tregs^73^, the constitutive co-expression of the IL-2 receptor (i.e. CD25) and OX40 may provide a basis for the Treg specificity of OX40L induced proliferation. Earlier studies have reported a decrease in Tregs in OX40^−/−^ mice and an increase in Tregs in OX40L over-expressing mice^74^ in support of our findings. Therefore, we used CD4^+^ T-cells from OX40^−/−^ mice in our G-BMDC co-cultures and confirmed that OX40L/OX40 interaction was required for Treg proliferation *ex vivo* (Fig. 1). Using NOD mice, we also established that OX40L treatment is capable of expanding functional Tregs *in vivo* (Fig. 2). Interestingly, using kinase inhibitors to identify signalling pathways critical for G-BMDC induced TCR-independent Treg proliferation we found potential involvement of NF-kB mediated signalling (Fig. 3).

Contrary to the widely accepted concept of TCR/TGF-β dependent origin of adaptive Tregs^75^, MHC class II-TCR-independent Treg generation and T-cell activation have been reported earlier^76^. Furthermore, TCR-independent activation of CD28^77^, the most characterized of the TCR-associated co-stimulatory molecules, has been reported to result in the enhancement of T-cell survival^78^ and cytokine production^79^. In the absence of TCR stimulation, CD28 stimulation is able to drive IKKα and a non-canonical NF-kB pathway leading to the activation of RelA/p52^80^. Echoing such an underlying mechanism, OX40 has been shown to associate with TRAF2 in a TCR-independent manner to form a signalosome along with PKC-Ѳ to activate NF-kB signalling and enhance T-cell survival and proliferation^33^.

We therefore used CD4^+^ T-cells from PKC-Ѳ deficient mice to verify if the above mechanism was responsible for our observed phenomenon. Although PKC-Ѳ^−/−^ Tregs were unable to proliferate robustly, they could be readily induced to proliferate by adding exogenous IL-2 (Fig. 4). Earlier studies have clearly shown the importance of PKC-θ signalling in T-cell activation and IL-2 production^81–83^. Consistent with these earlier observations, we also observed impaired IL-2 production from PKC-Ѳ^−/−^ Teff when compared to wild type Teff (Supplementary Fig. S3). These results suggest that PKC-Ѳ may not be directly involved in Treg proliferation, but may be responsible for the IL-2 secretion by Teffs that was required for Treg survival. While PKC-Ѳ^−/−^ Tregs showed proliferation comparable to that of WT Tregs, the OX40^−/−^ Tregs continued to show an intrinsic proliferation defect which could not be corrected by the addition of exogenous IL-2 (Fig. 5). Further, when we treated these mice with soluble OX40L, we found a robust increase in Treg percentages in both WT and PKC-Ѳ^−/−^ mice, but not in OX40^−/−^ mice (Fig. 6). These data suggested that OX40 ligation is critical for selective proliferation of Foxp3^+^ Tregs *in vivo* while PKC-Ѳ was dispensable. Therefore the role of PKC-Ѳ in the G-BMDC mediated Treg proliferation is primarily in the secretion of IL-2 by Teff that in turn acts as an essential survival factor for Tregs^73^.

Our data suggested that TCR independent OX40L/OX40 interaction likely activated NF-kB pathway (Fig. 3). It is pertinent to remember that a TCR-independent pathway has already been suggested to be involved in the development of thymic (or natural) Tregs^84, 85^. It has been suggested that self-reactive Treg precursors are rescued in the thymic medulla through a TCR-independent but IL-2R associated, STAT5 dependent mechanism^86^. To further investigate this possibility, we stimulated WT thymocytes with or without soluble OX40L in the presence of exogenous IL-2 for five days. We found that OX40L treatment induced significant (p < 0.005) increase in Treg proliferation compared to IL-2 alone (Supplementary Fig. S4a). Also, we found significant upregulation of pRelA and pRelB (Supplementary Fig. S4b). Furthermore, we found that OX40L treatment of thymocytes induced upregulation of RelA phosphorylation (Supplementary Fig. S4c). These data indicated that NF-kB activation was conserved between splenic and thymic Tregs as a mechanism of TCR-independent proliferation. Furthermore, we found pSTAT5 upregulation upon OX40L/IL-2 treatment compared to untreated cells (Supplementary Fig. S4d). It is pertinent to note that DCs have been shown to activate CD4^+^ T-cells to proliferate through a STAT5 dependent mechanism as well^87^. These reports suggest that although TCR-independent physiological mechanisms for both Foxp3^+^ and Foxp3^−^ CD4^+^ T-cell proliferation exist, Tregs may be more sensitive to these modalities of activation and proliferation. Thus, based on our data and available literature, it is possible that the convergence of NF-kB signaling through activation of OX40 by OX40L and STAT5 by IL-2 facilitates a Treg intrinsic program of proliferation independent of TCR-ligation. These possible mechanisms need to be fully elucidated in future studies.

Among the various TNF-family receptor members, we found elevated expression of OX40, TNFR2, 4-1BB and GITR mRNA in proliferating Tregs compared to control (Fig. 7). Interestingly, the genes encoding TNFR2, GITR, 4-1BB and OX40 are located on the same chromosome in both mouse and humans^88, 89^ and these same set of genes appear to be coordinately up regulated through TNFR2 activation^90^. Thus, the mechanisms involved downstream of OX40 activation in Tregs could be similar to those of well characterized TNFR2/4-1BB/GITR mediated mechanisms involved in Treg proliferation^22, 91^. While TRAF2/TRAF5 are generally associated with OX40 binding during T cell activation^24^; TRAF1 has been associated with TNFR2^92^ and 4-1BB^30, 33^; both leading to NF-kB activation. Indeed, we found elevated TRAF1 mRNA expression in proliferating Tregs compared to controls. Further, we found increased association of OX40 and TRAF1 in Tregs in the presence of OX40L. Thus our data suggests the possibility of a novel OX40/TRAF1 interaction that may be responsible for TCR-independent Treg proliferation.

OX40 was initially characterized as an effector T-cell co-stimulatory molecule and reported to enhance Teff cell proliferation upon TCR activation^19^. OX40 expression is predominantly confined to Tregs, but not conventional T-cells (Tconv) under steady state (Fig. 1b). The outcome of OX40L/OX40 interaction is context dependent and thus it can have pleotropic effects. OX40 expression is seen on Teff cells only upon their activation. Further, it has been characterized as a co-stimulatory molecule, which can enhance Teff cell proliferation upon TCR activation^19^. However, the precise role of OX40 signalling in Treg proliferation, function and Foxp3 expression remains unclear. Some recent studies have shown that OX40 signalling can enhance TCR-signal strength to facilitate Treg differentiation in the thymus^22^, while other studies have suggested a negative role in Treg function^55, 93^. Interestingly, the observed negative effect of OX40 signalling on Tregs was mainly due to exhaustion of IL-2 in the culture due to enhanced consumption by the proliferating Tregs, which could be readily reversed upon the addition of exogenous IL-2^55, 93^. Additionally, Tregs expanded using OX40 and IL-2R agonists were found to be potent suppressors and prevented graft rejection^94^. While these studies were carried out in the context of TCR signalling, we have identified a new pathway for the induction of Treg proliferation that is independent of canonical TCR signalling.

The mechanism of TCR-independent Treg proliferation has immense potential for clinical applications in conditions where Treg deficiency is implicated. Multiple autoimmune diseases in humans have been associated with reduced Treg populations. For example, reduced Treg numbers have been reported in patients with Sjorgen’s syndrome^95^, Systemic Lupus Erythematosus (SLE)^96, 97^ and T1D^98^. Therapeutic intervention through modulation of Tregs hold promise in the treatment of T-cell mediated autoimmune diseases through the restoration of immunological tolerance^99^. However, a method for specifically expanding Tregs *in vivo* without concomitant activation of Teff cells is still elusive. TCR-stimulation dependent protocol for *ex vivo* proliferation of polyclonal Tregs has been developed and is currently being used in clinical trials in T1D patients^100^. Such methods of adoptive transfer of Tregs has been found to be safe and efficacious^101^ but are cumbersome. Additionally such transfusion based methods require specialized clinical facility. There are however, several methods for selective Treg proliferation *in vivo* under development including low dose IL-2. This method relies on a lower IL-2 responsive threshold in Tregs than memory T-cells^102^. While this method does increase polyclonal Tregs *in vivo*, the therapeutic efficacy is yet to be demonstrated. Similar approaches using a combination of IL-2 and rapamycin did lead to a transient increase in Tregs *in vivo*, but eventually exacerbated the disease^9^. Thus, a reliable protocol for selective Treg proliferation *in vivo* that is safe and effective is yet to be developed. Our method has the potential to selectively expand Tregs *in vivo*, and thus complement some of the established methods by reducing the transfusion frequency, cost and complexity.

## Methods

### Mice

C57B6/j wild type, OX40^−/−^ (C57B6/j), and NOD mice were purchased from Jackson Laboratory. The MHC Class II^−/−^ (C57B6/j) mice were purchased from Taconic and PKC-Ѳ^−/−^ (C57B6/j) mice were kindly provided by Dr. Zuoming Sun, Department of Molecular Immunology, City of Hope, Duarte, CA. All mice were kept in an infection free environment in the Biological Resources Laboratory at the University of Illinois at Chicago. Food and water ad libitum was provided. All animal experiments were approved and performed in accordance with the guidelines set forth by the Animal Care and Use Committee at University of Illinois at Chicago. Soluble mouse OX40L used for the *in vivo* treatment was provided by Dr. Alan L. Epstein, Department of Pathology, University of Southern California Keck School of Medicine, Los Angeles, CA. 6 weeks-old NOD mice were treated i.p. with OX40L (200 µg/mouse/week). One week after the last treatment, mice were euthanized and organs harvested for analysis. For insulitis studies, control and treated mice were euthanized at 23-weeks age. C57B6/j, OX40-KO, and PKC-Ѳ-KO mice were intraperitoneally (i.p.) injected three times with OX40L (100 µg/mouse/week); one week after the last treatment mice were euthanized and organs harvested for analysis.

### T-cell isolation, G-BMDCs generation, and cell co-cultures

Total CD4^+^ T-cells and CD4^+^CD25^+^ T-cells were isolated using mouse CD4^+^ T-cell Isolation Kit, and mouse CD4^+^CD25^+^ Regulatory T-cell Isolation Kit, respectively (Miltenyibiotec Inc). G-BMDCs from both WT-BMDCs and MHC class II^−/−^ mice were generated as described before^13^. Briefly, bone marrow precursors isolated from femur and tibia bones were cultured in complete RPMI supplemented with 10% heat inactivated FBS in the presence of GM-CSF (20ng/ml). After 7 days, cells were harvested and used in the co-culture experiments. In co-culture experiments WT-BMDCs or MHC class II^−/−^ G-BMDCs and T-cells were mixed in a 1:2 ratio and incubated in complete RPMI containing 10% heat inactivated FBS. In co-culture experiments, cells were cultured for five days unless stated otherwise. In some experiments CD4+ T cells were stimulated with Mouse T-Activator CD3/CD28 Dynabeads® (life technologies).

### Flow cytometry, antibodies and intracellular staining

Samples were analysed using CyAn ADP analyser (Beckman Coulter). Anti-CD4-FITC, Anti-CD4-eFluor 780, Anti-Foxp3-PE, anti-Foxp3-PE-Cy5.5, anti-Foxp3-APC, anti-OX40-APC, anti-CD39-PE, and anti-OX40L-PE were purchased from Affymetrix eBioscience (San Diego, CA). Anti-phospho-p65-Alexa(R)488 and anti-phospho-RelB-PE were purchased from Cell Signaling Technology (Danvers, MA). Anti-CD44-FITC was purchased from Tonbo Biosciences (San Diego, CA). CellTrace violet used to detect cell proliferation was purchased from Thermo Fisher Scientific (Weltham, MA). Functional anti-CD3 was obtained from Tonbo Biosciences (San Diego, CA). For intracellular staining, cells were fixed and then permeabilized using Foxp3/Transcription Factor Staining Buffer Kit purchased from Tonbo Biosciences (San Diego, CA).

### Western blot

Thymocytes and splenic CD4+ T-cells were isolated and stimulated with IL-2, OX40L or both. After 24-72 h, cells were harvested, washed with PBS, lysed with Laemmli buffer (BioRad), and then resolved by SDS-PAGE. PVDF membrane (BioRad) was used for protein transfer and the membrane was blocked with a blocking solution (TBST, 5% skim milk, 3% BSA) overnight at 4 °C then washed. Blot was stained with an anti-pRelA primary antibody (Cell Signaling Technology) followed by a HRP-conjugated 2nd antibody, and then washed and developed using an ECL detection kit (Pierce Scientific).

### Screening of Kinase inhibitors

Total CD4^+^ or CD4^+^CD25^+^ T-cells were isolated from the spleen of NOD mice, incubated in 96 well plate containing Kinase Inhibitors: Lavendustin A (EGFRK), SB-202190 (p38 MAPK), GF 109203X (PKC), LY 294002 (PI3K), and SC-514 (IKK2) (final conc.12.5 µM) for 6 hours. Cells were then washed and co-cultured with G-BMDCs, Exogenous IL-2 was added in the case of CD4^+^CD25^+^ T-cells. After five days, cells were harvested and Treg proliferation was analysed by flow cytometry. Kinase inhibitors were obtained from High-Throughput Screening, Research Resources Center, University of Illinois at Chicago.

### *Ex vivo* suppression assay

Naïve CD4^+^ T-cells, depleted of CD4^+^CD25^+^ Tregs, were isolated from spleens of diabetic NOD mice using CD4^+^CD25^+^ Regulatory T-cell Isolation Kit from Miltenyibiotec Inc. Subsequently, these effector T cells were labelled with CellTrace violet from Thermo Fisher Scientific. Labelled cells were then incubated in round bottom 96-well plate containing anti-CD3 (2 ug/mL) and splenic APCs. Isolated CD4^+^CD25^+^ Tregs from OX40L-treated and untreated control NOD mice were co-cultured with CellTrace violet labelled naïve CD4^+^ T-cells at different ratios. After 48 hours, cells were harvested and analysed by flow cytometry for the proliferation of naïve CD4^+^ T-cells.

### Microscopy and ImageJ analysis

Pancreata were collected from euthanized mice, fixed in formalin, and embedded in paraffin. Tissue sections were prepared and stained using hematoxylin and eosin (H&E). Stained sections were evaluated to determine the degree of cellular infiltration into the islets. Alternatively, tissue sections were stained with guinea pig anti-insulin antibody (ab7842; abcam) followed by TRITC-conjugated anti-guinea pig IgG antibody (T7153) and DAPI (D9542) (Sigma-Adlrich). Sections were observed using a Zeiss Laser Scanning Microscope; LSM 710. Wild type (C57Bl/6) splenic CD4^+^ T cells were isolated as described above. Cells were then cultured in the presence of IL-2 (10 U/ml) with or without OX40L (7.5 µg/ml). After 48 hr, using Cytospin centrifuge, cells were transferred onto a glass slide. Cell were stained with anti-TRAF1-FITC antibody (sc-6253) and anti-Foxp3-Alexa Fluor 647 (sc-130666), anti-OX40 (sc-11403) purchased from Santa Cruz (Dallas, TX), -rabbit polyclonal antibodies followed by TRITC-conjugated Anti-Rabbit IgG antibody (ab6718), purchased from abcam (Cambridge, MA), and DAPI (D9542), purchased from Sigma-Adlrich (St. Louis, MO). After staining, slides were examined by confocal microscopy (Zeiss Laser Scanning Microscope; LSM 710). Confocal images were analysed with ImageJ software. Regulatory T cells were identified by Foxp3 and DAPI Staining. OX40 and TRAF1 staining was merged and the median intensity of merged colour from each cell was measured. Ten cells were analysed in the OX40L and IL-2 treated group and eleven were analysed in the IL-2 alone treated group. Statistical significance was measured between the two groups.

### RNA extraction, Real-Time-PCR, and Micro-array

Total RNA was extracted from freshly isolated Tregs (CD4^+^CD25^+^) and Teff (CD4^+^CD25^−^) using RNeasy column (Qiagen). cDNA synthesis was done using iScript cDNA synthesis kit (BioRad) following manufacturer’s instructions. iQ SYBER Green Supermix was used for RT-PCR on CFX Connect Real-Time PCR Detection System (BioRad). TRAF1-7 and IL-2  gene specific primers are listed in Supplementary Table S1. Fold change of gene expression values were calculated using comparative ΔCt method following normalization to GAPDH then expressed as fold change in Tregs over respective Teffs.

Microarray analysis of gene expression between proliferating and resting Tregs was done using the Affymetrix GeneChip Mouse Genome 430 2.0 array as described before^64^. Gene expression data was submitted to NCBI-GEO (Accession No. GSE81051)^64^. Heat maps for micro-array were generated using RStudio software.

### IL-2 measurement by ELISA and RT-PCR

WT and PKC-Ѳ^−/−^ splenic CD4^+^CD25^−^ T cells were isolated using mouse CD4^+^CD25^+^ Regulatory T-cell Isolation Kit (Miltenyibiotec Inc). Isolated cells were stimulated with Mouse T-Activator CD3/CD28 Dynabeads® (life technologies) for 20 hours. IL-2 levels in the culture supernatants were measured by ELISA using Mouse IL-2 ELISA Ready-SET-Go! (Invitrogen, Thermo Fisher Scientific). Cell pellets were used for RNA isolation, followed by cDNA synthesis. IL-2 mRNA levels were compared by RT-qPCR.

### Statistical analysis

MS-Excel from MS-Office application software was used to calculate average, standard deviation and statistical significance. For the determination of statistical significance Student’s T-test was used. A p-value of ≤0.05 was considered significant.

## Electronic supplementary material

Supplementary data

## References

[1] Abbas AK, Murphy KM, Sher A (1996). Functional diversity of helper T lymphocytes. Nature.

[2] Smith-Garvin JE, Koretzky GA, Jordan MS (2009). T cell activation. Annual review of immunology.

[3] Yamazaki S (2003). Direct expansion of functional CD25+ CD4+ regulatory T cells by antigen-processing dendritic cells. J Exp Med.

[4] Yu A (2015). Selective IL-2 responsiveness of regulatory T cells through multiple intrinsic mechanisms supports the use of low-dose IL-2 therapy in type 1 diabetes. Diabetes.

[5] Turner MS, Isse K, Fischer DK, Turnquist HR, Morel PA (2014). Low TCR signal strength induces combined expansion of Th2 and regulatory T cell populations that protect mice from the development of type 1 diabetes. Diabetologia.

[6] Hippen KL (2011). Massive *ex vivo* expansion of human natural regulatory T cells (T(regs)) with minimal loss of *in vivo* functional activity. Science translational medicine.

[7] Hoffmann P, Ermann J, Edinger M, Fathman CG, Strober S (2002). Donor-type CD4(+)CD25(+) regulatory T cells suppress lethal acute graft-versus-host disease after allogeneic bone marrow transplantation. J Exp Med.

[8] Shevach EM (2006). The lifestyle of naturally occurring CD4+ CD25+ Foxp3+. regulatory T cells. Immunological reviews.

[9] Long SA (2012). Rapamycin/IL-2 combination therapy in patients with type 1 diabetes augments Tregs yet transiently impairs beta-cell function. Diabetes.

[10] Brunstein CG (2011). Infusion of *ex vivo* expanded T regulatory cells in adults transplanted with umbilical cord blood: safety profile and detection kinetics. Blood.

[11] Putnam AL (2013). Clinical grade manufacturing of human alloantigen-reactive regulatory T cells for use in transplantation. Am J Transplant.

[12] Hoffmann P (2009). Loss of FOXP3 expression in natural human CD4+ CD25+ regulatory T cells upon repetitive *in vitro* stimulation. Eur J Immunol.

[13] Bhattacharya P, Gopisetty A, Ganesh BB, Sheng JR, Prabhakar BS (2011). GM-CSF-induced, bone-marrow-derived dendritic cells can expand natural Tregs and induce adaptive Tregs by different mechanisms. J Leukoc Biol.

[14] Baum PR (1994). Molecular characterization of murine and human OX40/OX40 ligand systems: identification of a human OX40 ligand as the HTLV-1-regulated protein gp34. The EMBO journal.

[15] Paterson DJ (1987). Antigens of activated rat T lymphocytes including a molecule of 50,000 Mr detected only on CD4 positive T blasts. Molecular immunology.

[16] Croft M (2010). Control of immunity by the TNFR-related molecule OX40 (CD134). Annual review of immunology.

[17] Hoshino A (2003). Critical role for OX40 ligand in the development of pathogenic Th2 cells in a murine model of asthma. European journal of immunology.

[18] Ito T (2004). Plasmacytoid dendritic cells regulate Th cell responses through OX40 ligand and type I IFNs. J Immunol.

[19] Gramaglia I, Weinberg AD, Lemon M, Croft M (1998). Ox-40 ligand: a potent costimulatory molecule for sustaining primary CD4 T cell responses. J Immunol.

[20] Rogers PR, Song J, Gramaglia I, Killeen N, Croft M (2001). OX40 promotes Bcl-xL and Bcl-2 expression and is essential for long-term survival of CD4 T cells. Immunity.

[21] Ruby CE (2009). Cutting Edge: OX40 agonists can drive regulatory T cell expansion if the cytokine milieu is right. J Immunol.

[22] Mahmud SA (2014). Costimulation via the tumor-necrosis factor receptor superfamily couples TCR signal strength to the thymic differentiation of regulatory T cells. Nat Immunol.

[23] Wajant H, Henkler F, Scheurich P (2001). The TNF-receptor-associated factor family: scaffold molecules for cytokine receptors, kinases and their regulators. Cellular signalling.

[24] Kawamata S, Hori T, Imura A, Takaori-Kondo A, Uchiyama T (1998). Activation of OX40 signal transduction pathways leads to tumor necrosis factor receptor-associated factor (TRAF) 2- and TRAF5-mediated NF-kappaB activation. The Journal of biological chemistry.

[25] So T, Croft M (2013). Regulation of PI-3-Kinase and Akt Signaling in T Lymphocytes and Other Cells by TNFR Family Molecules. Frontiers in immunology.

[26] Brenner D, Blaser H, Mak TW (2015). Regulation of tumour necrosis factor signalling: live or let die. Nat Rev Immunol.

[27] Prell RA (2003). OX40-mediated memory T cell generation is TNF receptor-associated factor 2 dependent. J Immunol.

[28] So T, Salek-Ardakani S, Nakano H, Ware CF, Croft M (2004). TNF receptor-associated factor 5 limits the induction of Th2 immune responses. J Immunol.

[29] Hauer J (2005). TNF receptor (TNFR)-associated factor (TRAF) 3 serves as an inhibitor of TRAF2/5-mediated activation of the noncanonical NF-kappaB pathway by TRAF-binding TNFRs. Proc Natl Acad Sci USA.

[30] Arch RH, Thompson CB (1998). 4-1BB and Ox40 are members of a tumor necrosis factor (TNF)-nerve growth factor receptor subfamily that bind TNF receptor-associated factors and activate nuclear factor kappaB. Molecular and cellular biology.

[31] Mestas J, Crampton SP, Hori T, Hughes CC (2005). Endothelial cell co-stimulation through OX40 augments and prolongs T cell cytokine synthesis by stabilization of cytokine mRNA. Int Immunol.

[32] Song J (2004). The costimulation-regulated duration of PKB activation controls T cell longevity. Nat Immunol.

[33] So T, Soroosh P, Eun SY, Altman A, Croft M (2011). Antigen-independent signalosome of CARMA1, PKCtheta, and TNF receptor-associated factor 2 (TRAF2) determines NF-kappaB signaling in T cells. Proc Natl Acad Sci USA.

[34] Baier G (1993). Molecular cloning and characterization of PKC theta, a novel member of the protein kinase C (PKC) gene family expressed predominantly in hematopoietic cells. The Journal of biological chemistry.

[35] Zhang EY, Kong KF, Altman A (2013). The yin and yang of protein kinase C-theta (PKCtheta): a novel drug target for selective immunosuppression. Advances in pharmacology.

[36] Meller N, Altman A, Isakov N (1998). New perspectives on PKCtheta, a member of the novel subfamily of protein kinase C. Stem cells.

[37] Meller N, Elitzur Y, Isakov N (1999). Protein kinase C-theta (PKCtheta) distribution analysis in hematopoietic cells: proliferating T cells exhibit high proportions of PKCtheta in the particulate fraction. Cellular immunology.

[38] Liu Y (2001). Protein kinase C theta is expressed in mast cells and is functionally involved in Fcepsilon receptor I signaling. J Leukoc Biol.

[39] Monks CR, Kupfer H, Tamir I, Barlow A, Kupfer A (1997). Selective modulation of protein kinase C-theta during T-cell activation. Nature.

[40] Yokosuka T (2008). Spatiotemporal regulation of T cell costimulation by TCR-CD28 microclusters and protein kinase C theta translocation. Immunity.

[41] Manicassamy S, Sadim M, Ye RD, Sun Z (2006). Differential roles of PKC-theta in the regulation of intracellular calcium concentration in primary T cells. Journal of molecular biology.

[42] Manicassamy S, Gupta S, Huang Z, Sun Z (2006). Protein kinase C-theta-mediated signals enhance CD4+ T cell survival by up-regulating Bcl-xL. J Immunol.

[43] Manicassamy S, Gupta S, Sun Z (2006). Selective function of PKC-theta in T cells. Cellular & molecular immunology.

[44] Kwon MJ, Wang R, Ma J, Sun Z (2010). PKC-theta is a drug target for prevention of T cell-mediated autoimmunity and allograft rejection. Endocrine, metabolic & immune disorders drug targets.

[45] Sun Z (2012). Intervention of PKC-theta as an immunosuppressive regimen. Frontiers in immunology.

[46] Gupta S (2008). Differential requirement of PKC-theta in the development and function of natural regulatory T cells. Molecular immunology.

[47] Baier-Bitterlich G (1996). Protein kinase C-theta isoenzyme selective stimulation of the transcription factor complex AP-1 in T lymphocytes. Mol Cell Biol.

[48] Cheatem D, Ganesh BB, Gangi E, Vasu C, Prabhakar BS (2009). Modulation of dendritic cells using granulocyte-macrophage colony-stimulating factor (GM-CSF) delays type 1 diabetes by enhancing CD4+ CD25+ regulatory T cell function. Clin Immunol.

[49] Vasu C, Dogan RN, Holterman MJ, Prabhakar BS (2003). Selective induction of dendritic cells using granulocyte macrophage-colony stimulating factor, but not fms-like tyrosine kinase receptor 3-ligand, activates thyroglobulin-specific CD4+/CD25+ T cells and suppresses experimental autoimmune thyroiditis. J Immunol.

[50] Sheng, J. R. *et al*. Suppression of experimental autoimmune myasthenia gravis by granulocyte-macrophage colony-stimulating factor is associated with an expansion of FoxP3+ regulatory T cells. *J Immunol* **177**, 5296–5306, doi:177/8/5296 [pii] (2006). 10.4049/jimmunol.177.8.5296 17015715

[51] Gaudreau S (2007). Granulocyte-macrophage colony-stimulating factor prevents diabetes development in NOD mice by inducing tolerogenic dendritic cells that sustain the suppressive function of CD4+ CD25+ regulatory T cells. J Immunol.

[52] Bernasconi E (2010). Granulocyte-macrophage colony-stimulating factor elicits bone marrow-derived cells that promote efficient colonic mucosal healing. Inflamm Bowel Dis.

[53] Gangi, E., Vasu, C., Cheatem, D. & Prabhakar, B. S. IL-10-producing CD4+ CD25+ regulatory T cells play a critical role in granulocyte-macrophage colony-stimulating factor-induced suppression of experimental autoimmune thyroiditis. *J Immunol* **174**, 7006–7013, doi:174/11/7006 [pii] (2005). 10.4049/jimmunol.174.11.7006 15905543

[54] Vu MD (2007). OX40 costimulation turns off Foxp3+ Tregs. Blood.

[55] Xiao X (2008). OX40/OX40L costimulation affects induction of Foxp3+ regulatory T cells in part by expanding memory T cells *in vivo*. J Immunol.

[56] Walker LST (2013). and CTLA-4: two intertwining pathways to immune tolerance. Journal of autoimmunity.

[57] Sakaguchi S, Yamaguchi T, Nomura T, Ono M (2008). Regulatory T cells and immune tolerance. Cell.

[58] Pan F (2009). Eos mediates Foxp3-dependent gene silencing in CD4+ regulatory T cells. Science.

[59] Li P (2015). CD39+ regulatory T cells attenuate allergic airway inflammation. Clinical and experimental allergy: journal of the British Society for Allergy and Clinical Immunology.

[60] Mandapathil M, Lang S, Gorelik E, Whiteside TL (2009). Isolation of functional human regulatory T cells (Treg) from the peripheral blood based on the CD39 expression. Journal of immunological methods.

[61] Wu C (2014). Galectin-9-CD44 interaction enhances stability and function of adaptive regulatory T cells. Immunity.

[62] Bollyky PL (2009). CD44 costimulation promotes FoxP3+ regulatory T cell persistence and function via production of IL-2, IL-10, and TGF-beta. J Immunol.

[63] Xiao X (2012). OX40 signaling favors the induction of T(H)9 cells and airway inflammation. Nat Immunol.

[64] Kumar P (2017). Soluble OX40L and JAG1 Induce Selective Proliferation of Functional Regulatory T-Cells Independent of canonical TCR signaling. Sci Rep.

[65] Iwanaga R (2008). Activation of the cyclin D2 and cdk6 genes through NF-kappaB is critical for cell-cycle progression induced by HTLV-I Tax. Oncogene.

[66] Santamaria D (2007). Cdk1 is sufficient to drive the mammalian cell cycle. Nature.

[67] Pfoertner S (2006). Signatures of human regulatory T cells: an encounter with old friends and new players. Genome biology.

[68] Ohshima Y (1998). OX40 costimulation enhances interleukin-4 (IL-4) expression at priming and promotes the differentiation of naive human CD4(+) T cells into high IL-4-producing effectors. Blood.

[69] So T, Song J, Sugie K, Altman A, Croft M (2006). Signals from OX40 regulate nuclear factor of activated T cells c1 and T cell helper 2 lineage commitment. Proc Natl Acad Sci USA.

[70] Kaur D, Brightling C (2012). OX40/OX40 ligand interactions in T-cell regulation and asthma. Chest.

[71] Maddur MS (2014). Human B cells induce dendritic cell maturation and favour Th2 polarization by inducing OX-40 ligand. Nat Commun.

[72] Ishii N, Takahashi T, Soroosh P, Sugamura K (2010). OX40-OX40 ligand interaction in T-cell-mediated immunity and immunopathology. Advances in immunology.

[73] Setoguchi R, Hori S, Takahashi T, Sakaguchi S (2005). Homeostatic maintenance of natural Foxp3(+) CD25(+) CD4(+) regulatory T cells by interleukin (IL)-2 and induction of autoimmune disease by IL-2 neutralization. J Exp Med.

[74] Takeda I (2004). Distinct roles for the OX40-OX40 ligand interaction in regulatory and nonregulatory T cells. J Immunol.

[75] Shevach EM, Tran DQ, Davidson TS, Andersson J (2008). The critical contribution of TGF-beta to the induction of Foxp3 expression and regulatory T cell function. Eur J Immunol.

[76] Scholzen A, Mittag D, Rogerson SJ, Cooke BM, Plebanski M (2009). Plasmodium falciparum-mediated induction of human CD25Foxp3 CD4 T cells is independent of direct TCR stimulation and requires IL-2, IL-10 and TGFbeta. PLoS pathogens.

[77] Raab M, Pfister S, Rudd CE (2001). CD28 signaling via VAV/SLP-76 adaptors: regulation of cytokine transcription independent of TCR ligation. Immunity.

[78] Marinari B, Costanzo A, Marzano V, Piccolella E, Tuosto L (2004). CD28 delivers a unique signal leading to the selective recruitment of RelA and p52 NF-kappaB subunits on IL-8 and Bcl-xL gene promoters. Proc Natl Acad Sci USA.

[79] Tuosto L (2011). NF-kappaB family of transcription factors: biochemical players of CD28 co-stimulation. Immunology letters.

[80] Porciello N, Tuosto L (2016). CD28 costimulatory signals in T lymphocyte activation: Emerging functions beyond a qualitative and quantitative support to TCR signalling. Cytokine & growth factor reviews.

[81] Werlen G, Jacinto E, Xia Y, Karin M (1998). Calcineurin preferentially synergizes with PKC-theta to activate JNK and IL-2 promoter in T lymphocytes. EMBO J.

[82] Coudronniere N, Villalba M, Englund N, Altman A (2000). NF-kappa B activation induced by T cell receptor/CD28 costimulation is mediated by protein kinase C-theta. Proc Natl Acad Sci USA.

[83] Wang XD (2015). TCR-induced sumoylation of the kinase PKC-theta controls T cell synapse organization and T cell activation. Nat Immunol.

[84] Burchill MA (2008). Linked T cell receptor and cytokine signaling govern the development of the regulatory T cell repertoire. Immunity.

[85] Liston A (2008). Differentiation of regulatory Foxp3+ T cells in the thymic cortex. Proc Natl Acad Sci USA.

[86] Pacholczyk R, Kern J (2008). The T-cell receptor repertoire of regulatory T cells. Immunology.

[87] Wang Y, Seidl T, Whittall T, Babaahmady K, Lehner T (2010). Stress-activated dendritic cells interact with CD4+ T cells to elicit homeostatic memory. Eur J Immunol.

[88] Birkeland ML, Copeland NG, Gilbert DJ, Jenkins NA, Barclay AN (1995). Gene structure and chromosomal localization of the mouse homologue of rat OX40 protein. European journal of immunology.

[89] Nocentini G (2000). Gene structure and chromosomal assignment of mouse GITR, a member of the tumor necrosis factor/nerve growth factor receptor family. DNA and cell biology.

[90] Hamano R, Huang J, Yoshimura T, Oppenheim JJ, Chen X (2011). TNF optimally activatives regulatory T cells by inducing TNF receptor superfamily members TNFR2, 4-1BB and OX40. European journal of immunology.

[91] Chen X, Baumel M, Mannel DN, Howard OM, Oppenheim JJ (2007). Interaction of TNF with TNF receptor type 2 promotes expansion and function of mouse CD4+ CD25+ T regulatory cells. J Immunol.

[92] Cabal-Hierro L (2014). TRAF-mediated modulation of NF-kB AND JNK activation by TNFR2. Cellular signalling.

[93] Vu MD (2007). OX40 costimulation turns off Foxp3(+) tregs. Blood.

[94] Xiao X (2012). New insights on OX40 in the control of T cell immunity and immune tolerance *in vivo*. J Immunol.

[95] Li X (2007). T regulatory cells are markedly diminished in diseased salivary glands of patients with primary Sjogren’s syndrome. The Journal of rheumatology.

[96] Crispin JC, Martinez A, Alcocer-Varela J (2003). Quantification of regulatory T cells in patients with systemic lupus erythematosus. Journal of autoimmunity.

[97] Miyara M (2005). Global natural regulatory T cell depletion in active systemic lupus erythematosus. J Immunol.

[98] Baecher-Allan C, Hafler DA (2006). Human regulatory T cells and their role in autoimmune disease. Immunological reviews.

[99] Sakaguchi S, Wing K, Miyara M (2007). Regulatory T cells - a brief history and perspective. European journal of immunology.

[100] Bluestone JA (2015). Type 1 diabetes immunotherapy using polyclonal regulatory T cells. Science translational medicine.

[101] Marek-Trzonkowska N (2014). Therapy of type 1 diabetes with CD4(+)CD25(high)CD127-regulatory T cells prolongs survival of pancreatic islets - results of one year follow-up. Clin Immunol.

[102] Rosenzwajg M (2015). Low-dose interleukin-2 fosters a dose-dependent regulatory T cell tuned milieu in T1D patients. Journal of autoimmunity.

