# Supplementary material for: PKC-ѳ is dispensable for OX40L-induced TCR-independent Treg proliferation but contributes by enabling IL-2 production from effector T-cells
Source: Sci Rep. 2017 Jul 26;7:6594. doi: 10.1038/s41598-017-05254-8 (PMC5529425; doi:10.1038/s41598-017-05254-8)
Supplement: Supplementary file 1 — Supplementary data [file 41598_2017_5254_MOESM1_ESM.doc]

**PKC-ϴ is dispensable for OX40L-induced TCR-independent Treg proliferation but contributes by enabling IL-2 production from effector T-cells**

Khaled Alharshawi^1^, Alejandra Marinelarena^1^, Prabhakaran Kumar^1^, Osama El-Sayed^1^, Palash Bhattacharya^1^, Zuoming Sun^2^, Alan L. Epstein^3^, Ajay V. Maker^1, 4^, and Bellur S. Prabhakar^1,*^

^1^Department of Microbiology and Immunology, University of Illinois College of Medicine, Chicago, Illinois; ^2^Department of Immunology, Beckman Research Institute, City of Hope, Duarte, CA.; ^3^Department of Pathology, University of Southern California Keck School of Medicine, Los Angeles, California; ^4^Department of Surgery, Division of Surgical Oncology, University of Illinois College of Medicine, Chicago, Illinois.

*** Corresponding Author:**

Bellur S. Prabhakar, MSc. PhD

Professor

Departments of Microbiology and Immunology, and Ophthalmology

Associate Dean for Technological Innovation and Training

University of Illinois College of Medicine

Room E-705, (M/C 790)

835 S. Wolcott Ave

Chicago, IL 60612, USA

Tel: +1-312-996-4945

FAX: +1-312-996-6415

bprabhak@uic.edu

**Supplementary Figures**

**Supplementary Fig-S1**

**
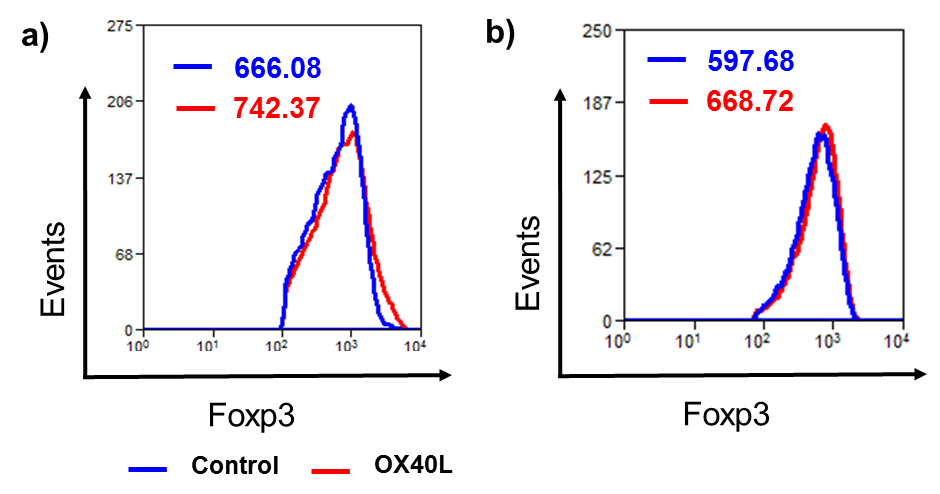
**

**Supplementary Fig. S1: OX40L expanded Tregs do not lose Foxp3 Expression.** A) Splenic CD4^+^ T-cells were cultured with soluble OX40L in the presence of IL-2 for 5 days. Foxp3 expression was compared between freshly isolated control and OX40L treated Tregs. Overlay histograms show representative MFI values between control (Blue) vs OX40L (Red) treated Tregs. B) C57BL6 mice were treated with or without OX40L once a week for 3 weeks. Foxp3 expression in splenic Tregs was compared between control and OX40L treated mice. Overlay histograms show representative MFI values between control (Blue) vs OX40L (Red) treated mice.

**Supplementary Fig. S2**


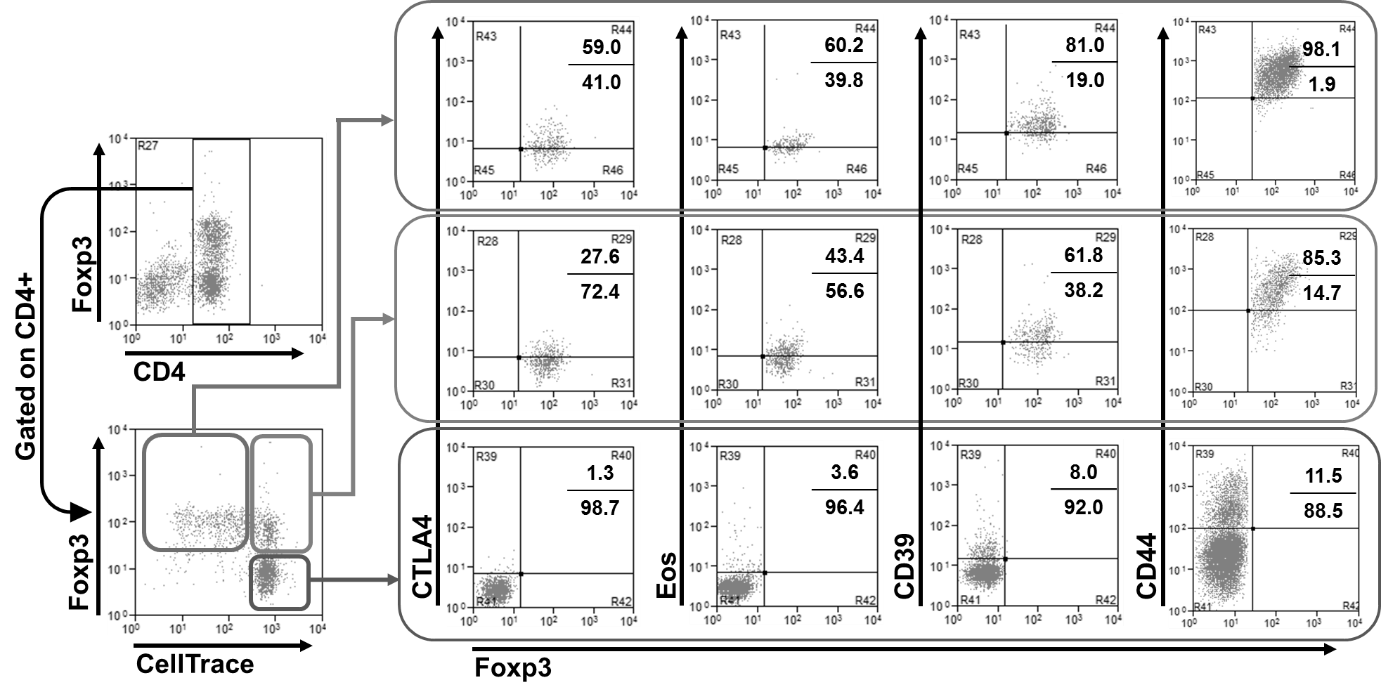


**Supplementary Fig. S2: Proliferating Tregs exhibit robust expression of molecules associated with suppressive function.** Total CD4^+^ T-cells from spleens of NOD mice were isolated, labelled with CellTrace violet and then co-cultured with G-BMDCs. On day 5 of co-culture, cells were harvested and analysed by flow cytometry for expression of Treg related molecules.

**Supplementary Fig. S3**

**Supplementary Fig. S3: Impaired IL-2 production in PKC-θ^-/-^ T-cells.** WT and PKC-θ^-/-^ CD4+T-cells were stimulated with anti-CD3/CD28 overnight. A) Post treatment mRNA expression of IL-2 was analysed by RT- qPCR and results were expressed as fold induction over WT. B) IL-2 secretion was analysed by ELISA and bar graphs represent levels of IL-2 in the culture supernatants. (Values represent average ± SD. * =P<0.05 and *** = p<0.0005 vs WT).

**Supplementary Fig. S4**


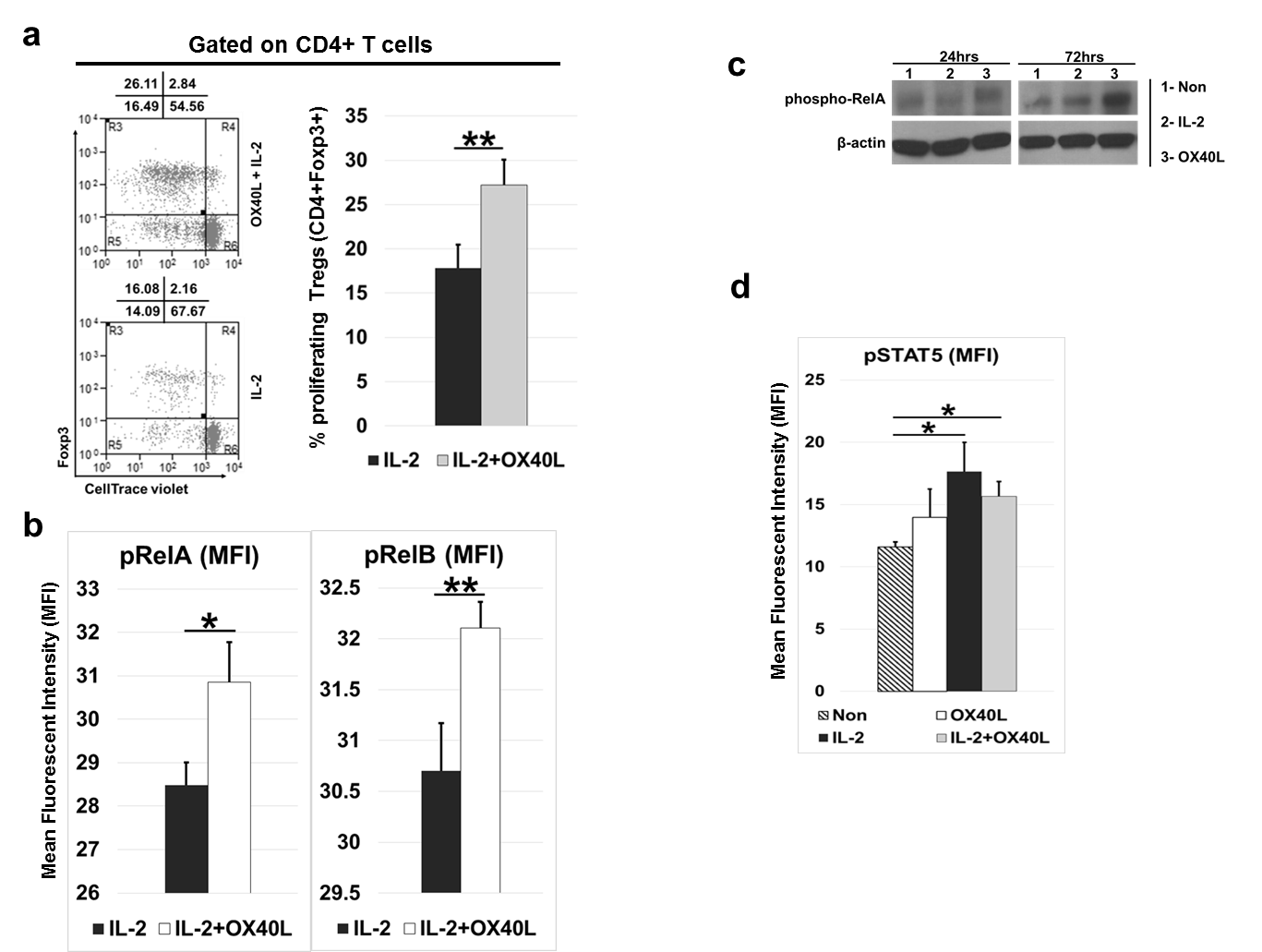


**Supplementary Fig. S4: NF-kB and STAT5 pathways converge in thymic Treg proliferation induced by G-BMDCs.** A & B) Thymocytes from WT mice were CellTrace violet labelled and cultured in the presence of IL-2 with or without OX40L for 5 days. Cells were harvested and analysed by flow cytometry. A) Representative dot plots (left) and summary bar graphs (right) showing the percentage of Tregs proliferating. B) Bar graphs showing the Mean Fluorescent Intensity (MFI) of pRelA (left) and pRelB (right) in Tregs (CD4+Foxp3+ T cells). C) Western blot analysis of pRelA activation by OX40L or IL-2 ex vivo in thymocyte cultures. D) Activation of STAT5 in thymocyte cultures in the presence of no stimuli, IL-2 alone, OX40L alone, or IL-2 + OX40L for 24hrs. Cells were harvested and analysed by flow cytometry for pSTAT5 using Mean Fluorescent Intensity (MFI), cells were gated on Tregs (CD4+Foxp3+ T-cells). Values showing average ± SD, * =P<0.05, ** = p<0.005, and *** = p<0.0005.

**Supplementary Tables**

| **Supplementary Table S1: Primers sequence for quantitative RT-PCR** | | |
| --- | --- | --- |
| **Target gene** | **primer** | **Sequence** |
| **TRAF1** | Forward | 5’- AGG GTG GTG GAA TTA CAG CAA -3' |
|  | Reverse | 5’- GCA GTG TAG AAA GCT GGA GAG -3' |
| **TRAF2** | Forward | 5’-AGA GAG TAG TTC GGC CTT TCC -3' |
|  | Reverse | 5’- GAG CAT CCA TCA TTG GGA CAG -3' |
| **TRAF3** | Forward | 5’-AGA CCC GAG AAA ACC GTC G -3' |
|  | Reverse | 5’- CTC CTT TTG AGG AAC TGT AGC TG -3' |
| **TRAF4** | Forward | 5’- CCC GGC TTC GAC TAC AAG TTC-3' |
|  | Reverse | 5’- TCA GGG CAT TTG AAG ACT CCT-3' |
| **TRAF5** | Forward | 5’- TTT GAG CCC GAC ACC GAG TA -3' |
|  | Reverse | 5’- AGA GAC CGG ATG CAC TGC T -3' |
| **TRAF6** | Forward | 5’- AAA GCG AGA GAT TCT TTC CCT -3' |
|  | Reverse | 5’- ACT GGG GAC AAT TCA CTA GAG C -3' |
| **TRAF7** | Forward | 5’- GAC CAG GAT GGA AAC AAC CTT T -3' |
|  | Reverse | 5’- AAT AGG CAA GGG TGC TAG AGG -3' |
| **IL-2** | Forward | 5’- TGA GCA GGA TGG AGA ATT ACA GG-3’ |
|  | Reverse | 5’- GTC CAA GTT CAT CTT CTA GGC AC-3’ |
